# Development and Validation of Non-Invasive Machine-Learning Screening Models for Pediatric Malnutrition in Hospitalized Children: A Single-Center Study

**DOI:** 10.3390/children13050617

**Published:** 2026-04-29

**Authors:** Petra Klanjšek, Petra Povalej Bržan, Nataša Marčun Varda, Mirjam Močnik, Sonja Golob Jančič, Miha Kovačič, Majda Pajnkihar

**Affiliations:** 1 Faculty of Health Sciences, University of Maribor, Žitna ulica 15, 2000 Maribor, Slovenia; majda.pajnkihar@um.si; 2 Faculty of Medicine, University of Maribor, Taborska ulica 8, 2000 Maribor, Slovenia; petra.povalej@um.si (P.P.B.); natasa.marcunvarda@ukc-mb.si (N.M.V.); 3 Faculty of Electrical Engineering and Computer Science, University of Maribor, Koroška cesta 46, 2000 Maribor, Slovenia; 4 University Division of Paediatrics, University Medical Centre Maribor, Ljubljanska ulica 5, 2000 Maribor, Slovenia; mirjam.mocnik@ukc-mb.si (M.M.); sonja.golobjancic@ukc-mb.si (S.G.J.); 5 Faculty of Mechanical Engineering, University of Ljubljana, Aškerčeva cesta 6, 1000 Ljubljana, Slovenia; miha.kovacic@store-steel.si; 6 College of Industrial Engineering, Mariborska cesta 2, 3000 Celje, Slovenia; 7 Štore Steel d.o.o., Železarska cesta 3, 3220 Štore, Slovenia

**Keywords:** pediatric malnutrition, pediatric undernutrition, nutritional screening tools, nutritional risk screening/assessment, machine learning, evolutionary computation, genetic programming, malnutrition risk screening, nutritional assessment tool, SGNA

## Abstract

**Highlights:**

**What are the main findings?**

- We developed and validated new pediatric malnutrition screening models based on non-invasive indicators; the best-performing models (GP, ANN, and ANFIS) showed high apparent diagnostic accuracy in this dataset.
- Simplified decision tree models showed lower accuracy but offered greater transparency and feasibility for routine ward-level use.

**What are the implications of the main findings?**

- Machine learning and evolutionary approaches show strong potential to improve pediatric screening of risk of malnutrition.
- These models show potential as supportive tools in settings where a full subjective malnutrition assessment is not feasible, but further external validation is required before clinical implementation.

**Abstract:**

**Background/Objectives**: Child malnutrition is a global health challenge linked to poor growth, impaired development, weakened immunity, and adverse outcomes. Early risk detection is essential, but current screening tools differ in accuracy and feasibility. This study aimed to develop and validate new bedside pediatric malnutrition screening models based on machine learning and evolutionary computation methods that can capture complex patterns in non-invasive clinical indicators while remaining practical for routine ward use. **Methods**: We conducted a cross-sectional study including 180 hospitalized children (1 month–18 years) recruited consecutively from six pediatric wards. The required sample size (minimum 138 participants) was calculated a priori using national prevalence estimates of pediatric undernutrition (4–9.5%) to ensure adequate precision at a 95% confidence level. Data collection included a questionnaire, anthropometry, subjective malnutrition risk assessment, and the Subjective Global Nutritional Assessment (SGNA) tool. Screening models were developed using decision trees, random forests, XGBoost, lasso regression, artificial neural networks, ANFIS, and genetic programming. Their performance was evaluated against the SGNA tool and physician-based subjective malnutrition risk assessment using sensitivity, specificity, AUC, and Cohen’s κ. **Results**: Machine learning and intelligent evolutionary models (GP, ANN, and ANFIS) showed the best performance in this sample, with substantial to high agreement (κ = 0.81–1.00) and high diagnostic accuracy (AUC = 0.92–1.00) with the subjective malnutrition risk assessment. The GP model demonstrated the highest apparent accuracy in this dataset, but also higher complexity, whereas simpler models such as decision trees showed lower accuracy but greater interpretability and feasibility for routine clinical use. However, validation was performed on a relatively small independent sample, and no external validation was conducted, which may limit the generalizability of the findings. **Conclusions**: While complex models may serve as digital assessment instruments, simpler models are rapid and more suitable for bedside screening. All developed models are non-invasive and cost-effective and show potential for supportive approaches for early detection of malnutrition risk at hospital admission. However, given the limited validation sample and the absence of external validation, these findings should be interpreted with caution, and further large-scale, multicenter studies are required to confirm generalizability and clinical applicability.

## 1. Introduction

Childhood malnutrition is a global challenge. Worldwide, 149 million children under five are stunted and 45 million are wasted. These conditions arise from food scarcity, inadequate nutrition, poverty, and social inequalities [1,2]. The reported prevalence of pediatric malnutrition in outpatient and inpatient settings ranges from 2.5% to 51% [3]. This reflects differences in diagnostic definitions, applied assessment criteria, and the growth charts [4]. In Slovenia, undernutrition prevalence among primary school children ranges from 4.7% to 9.5% depending on sex, while studies in tertiary hospitals report rates of around 40% in hospitalized children across various diagnoses [5].

Adequate and balanced nutrition in children is essential for normal growth, neurocognitive development, and immune function [6,7,8,9,10]. Malnutrition, most commonly undernutrition (wasting, stunting, and underweight), is associated with inflammation [10], and leads to impaired growth, weakened immunity, reduced cognitive potential, and increased susceptibility to infections [2,10,11,12]. According to the World Health Organization, malnutrition also includes inadequate intake of vitamins or minerals, as well as overweight, obesity, and diet-related noncommunicable diseases [2]. In hospitalized children, it is also linked to adverse outcomes, including prolonged hospital stay, increased healthcare costs, and higher mortality [2,13,14].

Although several methods exist to assess nutritional status, traditional anthropometric classifications such as the Gomez and the Waterlow systems are limited because they rely exclusively on anthropometric measures. Anthropometric indices may underestimate the true prevalence of malnutrition [15]. These limitations extend to currently used pediatric screening tools and WHO growth-monitoring standards. In hospitalized children, the use of WHO standards is often constrained by practical measurement challenges, particularly when accurate height or length cannot be obtained, reducing the reliability of anthropometry-based assessment [16,17,18,19]. Existing screening tools vary in their construction, cut-off values, and reference standards, often include subjective items, and show wide variability in sensitivity and specificity [4,20]. Consequently, they may miss mild or early malnutrition and often fail to account for etiological or clinical factors relevant in inpatient settings [17,20].

Early detection of malnutrition is crucial for improving pediatric care and outcomes through targeted nutritional interventions [13]. International guidelines (ASPEN, ESPEN, ESPGHAN) emphasize the importance of systematic screening at hospital admission to ensure appropriate nutritional assessment and treatment [21,22,23]. However, no universally accepted gold standard for pediatric nutritional screening exists, and currently available tools remain limited in their diagnostic accuracy and clinical applicability across different inpatient settings [4].

Recent advances in machine learning and evolutionary computation methods have shown promise in developing screening tools with high diagnostic accuracy, as already demonstrated in disease prediction and medical diagnosis [24]. However, their application in pediatric nutritional screening remains limited, particularly in approaches that combine non-invasive clinical indicators with advanced analytical methods for bedside use.

Although several pediatric nutritional screening tools exist, they are predominantly rule-based instruments that rely on fixed scoring systems, assume largely linear relationships between indicators, and often depend on anthropometric cut-offs or subjective items. As shown in recent validation studies, their diagnostic performance varies substantially across inpatient settings, with sensitivity frequently too low to detect early or mild malnutrition, and specificity too variable for consistent clinical use [4]. Moreover, our previously developed SPNRS tool, while improving feasibility at the bedside, still required predefined weights and could not fully account for complex, non-linear interactions between clinical indicators [5]. Together, these limitations highlight the need for more flexible, data-driven approaches that integrate multiple non-invasive clinical features and optimize their contribution to risk prediction, thereby improving accuracy without increasing clinical workload.

Machine learning and evolutionary computation methods are well suited to this challenge because they can model non-linear relationships, detect interactions among heterogeneous clinical indicators, and derive prediction rules empirically rather than relying solely on fixed a priori weights or cut-offs. In addition, these approaches enable comparison between models with different levels of complexity, allowing both the identification of high-performing predictive models and the selection of simpler, more interpretable models for routine bedside use [25,26,27]. This methodological approach is therefore directly aligned with the identified limitations of existing pediatric malnutrition screening methods.

The rationale for this study was therefore to address this gap by developing and validating novel screening models based on machine learning and evolutionary computation that are rapid, simple, non-invasive, and feasible for routine bedside use. The significance of this work lies in its potential to improve early detection of malnutrition, reduce variability in screening practice, and support more standardized, evidence-based institutional and national policies for pediatric nutritional risk screening.

This study aimed to develop and validate bedside screening models for detecting malnutrition risk in pediatric patients (aged 1 month to 18 years) using machine learning and evolutionary computation methods. Consistent with usability criteria, the models were intended to be rapid, simple, non-invasive, cost-effective, and feasible for ward-level use with minimal staff training [28]. The models were validated against reference standards, including subjective malnutrition risk assessment and the Subjective Global Nutritional Assessment (SGNA).

## 2. Materials and Methods

### 2.1. Design

We conducted a cross-sectional study in two phases between May and October 2021 (Figure S1 in Supplementary Material S1) as previously described in detail [5]. The development dataset was collected from May to July 2021, and the validation dataset was collected from September to October 2021. Computational model development and training were performed retrospectively in September 2025 using the previously collected 2021 development dataset. Data collection combined three approaches: (1) a questionnaire, (2) a subjective malnutrition risk assessment, and (3) the SGNA tool. Insights from the qualitative phase informed the quantitative analyses [29].

### 2.2. Ethics Approval and Ethical Considerations

The study was approved by the National Medical Ethics Committee of the Republic of Slovenia (0120-329/2016-3 KME 40/07/1), with additional site-specific approval from the participating hospital. Written and verbal information about the study was provided to parents or legal guardians, and written informed consent was obtained prior to enrollment [30]. In addition, age-appropriate information about the study was provided to children, and their willingness to participate was respected. Children were included in the study only if both the child and their parent or legal guardian agreed to participate. If either the child or the parent/legal guardian declined participation, the child was not enrolled in the study.

### 2.3. Participants

The study team included researchers from the Faculty of Health Sciences, the Faculty of Medicine, and the pediatric clinic. Sample size was determined using prevalence data from the SLOFIT study [31]. Based on an estimated malnutrition prevalence of 4–9.5% among school-aged children, a minimum of 138 patients was required, assuming a 10% prevalence difference and 95% confidence interval. A detailed description of the study population and the nutritional characteristics of the children included has been published previously [5] and is therefore not repeated here.

Children aged 1 month to 18 years who were admitted to pediatric wards with an anticipated hospital stay of more than 24 h were eligible for inclusion. The 24 h requirement ensured that all study procedures (questionnaire, anthropometry, subjective assessment, and SGNA) could be completed reliably, as shorter admissions do not provide sufficient time or consistent parental availability for accurate data collection.

This age range was chosen because, in our institution, patients up to 18 years of age are routinely admitted to pediatric wards and managed according to pediatric nutrition guidelines. Although the WHO classifies individuals aged 10–19 years as adolescents [32], in the context of hospital organization and clinical practice they form an integral part of the pediatric population for which bedside nutritional screening tools are required. Our aim was therefore to develop models that are applicable to all patients cared for on pediatric wards, including infants, children, and adolescents. Neonates younger than 1 month were not included. In our setting, this age group is treated almost exclusively in dedicated neonatal units or intensive neonatal care units, where clinical characteristics, feeding patterns, and growth references differ substantially from those of older infants. In addition, key study procedures and reference standards (questionnaire items, subjective malnutrition risk assessment, and SGNA) have not been validated for neonates and were not considered appropriate for this population. For these reasons, and to ensure a clinically homogeneous target group for the development of bedside screening models, we restricted inclusion to children aged 1 month to 18 years. Enrollment was conducted consecutively from six distinct clinical wards if data collection was possible within the first 24 h after admission.

Participation was limited to children whose legal guardians provided written informed consent and who themselves agreed to participate in an age-appropriate manner. Children treated exclusively in the emergency department, outpatient clinics, neonatal units, or intensive care units were not eligible, because these settings differ substantially from general pediatric wards in terms of diagnostic spectrum, length of stay, parental availability, and clinical stability [30]. In these contexts, the full study protocol (questionnaire, anthropometric measurements, subjective malnutrition risk assessment, and SGNA) could not be implemented in a standardized and feasible way.

### 2.4. Development and Validation of Nutritional Screening Models

#### 2.4.1. Qualitative Data

To identify non-invasive indicators relevant to pediatric malnutrition in hospitalized settings, we conducted an extensive literature review. This phase was informed by published validation studies of pediatric nutritional screening tools [4]. The extensive literature review was carried out in PubMed, CINAHL, and MEDLINE. Using combinations of keywords related to pediatric malnutrition, hospitalized children, and undernutrition, we constructed a comprehensive search strategy. Boolean operators AND and OR were used to generate the following search string: (“premature*” OR “immature*” OR “child*” OR “baby” OR “infant*” OR “newborn*” OR “neonate*” OR “kid*” OR “babies” OR “adolescent*” OR “teenager*”) AND (“undernutrition*” OR “undernourish*” OR “malnutrition*” OR “malnourish*”) AND (hospital*).

Only peer-reviewed original studies, clinical guidelines, and validation studies of pediatric screening/assessment tools were included. We included in the analysis and synthesis all English-language articles addressing malnutrition in hospitalized children and adolescents, specifically those reporting etiologic factors, clinical consequences, or prevalence of malnutrition. No restrictions were applied regarding publication date. Studies conducted in low- and middle-income countries were excluded. Grey literature was not systematically searched.

Two researchers independently screened and selected the literature: a doctoral-level pediatric nurse researcher and a pediatrician. Disagreements were resolved through discussion with a senior pediatrician. Using inductive content analysis [33], we derived etiologic determinants and clinical and functional consequences of malnutrition from the included studies. These findings, together with evidence from a systematic review of the criteria employed in 24 existing pediatric screening tools [5], informed the initial pool of items for the questionnaire. Following the recommendations of Streiner et al. [34] the items were translated bidirectionally between English and Slovene.

When selecting candidate risk-assessment features, we also considered the ASPEN Pediatric Nutrition Care Pathway and the Academy of Nutrition and Dietetics (AND)/ASPEN pediatric malnutrition criteria [35,36].

A multidisciplinary expert panel of pediatricians (*n* = 3) and nurses (*n* = 6), from the pediatric clinic and Faculty of Health Sciences, reviewed the initial questionnaire for face validity, clarity, and consistency with professional terminology [34]. Suggested revisions were adopted where needed. The questionnaire items were developed based on an extensive literature review and multidisciplinary expert review for face validity and clarity. The questionnaire was not formally pilot tested in an independent sample prior to the main data collection; therefore, questionnaire reliability coefficients (e.g., test–retest or internal consistency) are not available. Data collection followed a standardized protocol and assessors were trained. The reported inter-rater agreement (κ = 0.70–1.00; Supplementary Table S2) relates to the physician-based reference assessments, not to the questionnaire items.

The finalized questionnaire contained 94 items in thematic domains (demographics and admission details, medical diagnosis, anthropometry, clinical signs, nutritional intake, gastrointestinal symptoms, psychological factors, body temperature, tissue resistance and regenerative capacity, physical endurance, socio-emotional functioning, and menstrual status), yielding 277 variables due to multiple-choice responses, as previously described in detail [5].

#### 2.4.2. Development of Nutritional Screening Models: Quantitative Data

Data collection followed previously published procedures [5,30]. During the development phase (*n* = 142 children) in May and July 2021, a doctoral-level research nurse conducted questionnaire interviews with parents (and children when appropriate). The physicians were blinded to the interview results. Anthropometric measurements (weight (W), height (H)/length (L), and mid-upper arm circumference (MUAC)) were obtained following WHO standards [37,38]. Body mass index (BMI) was calculated using PediTools (CDC Growth calculator for 0 to 36 months and CDC Growth calculator for 2 to 20 years) [39] and WHO Anthro software version 3.2.2 [40]. Nutritional status was classified according to ASPEN and Academy of Nutrition and Dietetics recommendations [35] using z-scores for weight-for-height/length (WFH/L), BMI, height-for-age (HFA), and MUAC. Nutrition status in children younger than two years were evaluated using the WHO Anthro program (Version 3.2.2, January 2011) [40]. For children two years and older, z-scores were evaluated using CDC 2000 growth reference charts were applied through the PediTools [39]. In the assessment of pediatric malnutrition, the diagnosis was established using a single criterion [41].

In parallel, research pediatricians conducted a subjective malnutrition risk assessment and SGNA tool evaluation, blinded to the nurse’s data. For model development, physician assessments were dichotomized into “not at risk” (normal, overweight, obese) and “at risk” (low, moderate, severe) of malnutrition. The SGNA tool was categorized as well-nourished, moderately malnourished, or severely malnourished [42]. Inter-rater reliability between research pediatricians was calculated in the first 80 children (κ = 0.7–1.0), indicating substantial to almost perfect agreement (Table S1 in Supplementary Material S2). Although participant recruitment for the development phase was conducted in 2021, computational model development and training were subsequently performed in January 2024 using the previously collected dataset. No additional participants were recruited in 2024; only model construction and training were performed using the original 2021 development dataset.

#### 2.4.3. Validation of Nutritional Screening Models: Quantitative Data

In September and October 2021 children were enrolled for model validation. Parents provided written consent. A research nurse performed screening, while a pediatrician independently carried out a subjective malnutrition risk assessment first and then the evaluation with SGNA tool. Both assessors were blinded to each other’s results. The protocol mirrored the procedures used in the Development of Nutritional Screening Models phase. Validation of the models was conducted in accordance with the recommendations of Klanjsek et al. [4,5]. Validation was performed using an independent hold-out sample collected in a separate study phase. The development dataset (*n* = 142) was used for model construction, and the validation dataset (*n* = 38) was used only for independent evaluation of model performance. This strategy was chosen to test model performance on previously unseen cases within the constraints of the available prospective single-center dataset.

Criterion validity was assessed by comparing the newly developed screening models with the subjective malnutrition risk assessment, which served as the primary reference standard for model validation. Concurrent validity was assessed against the SGNA tool, which served as the secondary reference standard for model validation. The physician-based assessment was chosen as the primary reference standard because it integrates expert judgment, medical history, anthropometric measurements, and physical examination, whereas SGNA was included as a structured and internationally recognized assessment tool to enable comparison with an established approach [5]. On the same day, the research nurse administered the new screening model, while the physician performed both the SGNA tool and the subjective malnutrition risk assessment on the same child.

### 2.5. Data Analysis

Prior to model development, the dataset was checked for completeness, consistency, and plausibility. Categorical variables were appropriately encoded for statistical and machine learning analyses, and continuous variables were inspected for outliers and distributional characteristics.

Feature selection was performed using a two-stage approach to reduce dimensionality and improve model stability. In the first stage, variables significantly associated with malnutrition risk were identified using Pearson’s χ^2^ test (*p* < 0.05), ensuring statistical relevance. In the second stage, the most informative predictors were selected using Random Forest variable importance, based on the Mean Decrease Accuracy metric. A threshold of >2 was applied to retain variables with the strongest predictive contribution.

This approach allowed the reduction in the initial high-dimensional dataset (277 variables) to a manageable set of predictors while balancing statistical significance and predictive performance. By limiting the number of input variables, the risk of overfitting was reduced, and model stability across different modelling techniques was improved. No additional penalization methods or formal dimensionality reduction techniques were applied, as the goal was to retain clinically interpretable variables. The combined use of statistical filtering and model-based feature importance aimed to ensure both relevance and robustness of selected predictors. By reducing the predictor space before model training, this approach decreased model variance and improved stability across different modelling techniques, as reflected in the consistent performance of models in the validation phase.

Analyses were performed using ISPSS (Version 28.0., IBM Corp: Armonk, NY, USA) [43], the R programming language in the RStudio environment, version 4.5.2 [44], MATLAB version 24.1 (The MathWorks Inc., Natick, MA, USA) [45], and Modern Visual AutoLISP (2020s generation) in AutoCAD LT 2024 [46]. Descriptive statistics were expressed as medians with 95% confidence interval (CI) or means with standard deviations (SD) for numerical variables, and frequencies with percentages for categorical variables. Anthropometric indices expressed as z-scores (WFA, HFA, BMI, MUAC, and TSF) were summarized as mean ± SD overall and within nutritional-risk groups (“Not at risk” vs. “At risk”). Between-group differences were assessed with Welch’s two-sample t-test (unequal variances); we report the mean difference (Not at risk − At risk) with its standard error and 95% confidence interval (CI).

The association between questionnaire variables and subjective malnutrition risk assessment was tested using Pearson’s χ^2^ test, with *p*-values < 0.05 considered statistically significant. Variable importance was evaluated with the Random Forest model; 30 variables with a Mean Decrease Accuracy value >2 were retained for the models’ development. To ensure an interpretable and clinically feasible number of predictors, we selected a threshold of Mean Decrease Accuracy >2, which represented a substantial separation from the highest observed Mean Decrease Accuracy value (12.7). This approach allowed retention of the strongest predictors while minimizing the risk of excluding clinically relevant variables.

Several machine learning and statistical models were developed. For clarity, the evaluated approaches were grouped into: (i) simpler and more interpretable models (decision trees and lasso regression); and (ii) more complex models capable of capturing higher-order non-linear relationships (random forest, XGBoost, ANN, ANFIS, and GP). Decision trees were constructed using different criteria: the Gini Index, the ID3 algorithm based on information Gain, and CHAID, which used Pearson’s χ^2^ test with a maximum depth of 3, at least 10 cases per parent node, and five cases per child node. The Random Forest (basic model with default parameters) and Random Forest (with optimized parameters) models were developed using the random forest algorithm. Model parameters included classification mode, 500 decision trees, and random selection of four variables at each node. Variable importance was assessed using the Mean Decrease in Gini. XGBoost model was implemented with all 30 variables, using a learning rate of 0.1, a maximum tree depth of 15, 25 trees, a subsample of 0.5, a colsample of 0.5, and logistic regression as the objective function. Lasso and Ridge regression models were developed using *glmnet* package (alpha = 1). The optimal λ was selected through cross-validation by minimizing mean squared error (MSE) (lambda = cv$lambda.min). Genetic programming was based on arithmetic operators (addition, subtraction, multiplication, division) as function genes and 30 input variables as terminal genes. Evolutionary settings included a population size of 1000, a maximum of 100 generations, reproduction probability of 0.2, crossover probability of 0.7, initial model depth between 2 and 20, maximum offspring depth after crossover of 30, and tournament size of 7. Artificial neural network (ANN) models were developed in Matlab and RStudio environment. The Matlab ANN was a pattern recognition network with 30 inputs, two outputs, and a single hidden layer of five neurons using the hyperbolic tangent sigmoid activation function (Tansig) (Figure S2, Supplementary Material S3). Training was performed with a gradient learning rate of 0.1, a maximum of 100 iterations, and the scaled conjugate gradient backpropagation algorithm (Trainscg). In RStudio environment, a Neural network (basic) model was constructed with 30 variables and three hidden layers of five neurons in each layer, while a Neural network (optimized) was developed using the eight most statistically significant variables identified by Pearson χ^2^ test. The settings included one hidden layer with two neurons.

The ANFIS model was built in the ANFIS editor using a data matrix of 31 columns and 142 rows, with 30 developed variables and last, binary target column, indicating nutritional status (1 = high risk of malnutrition). Initialization was performed using the subtractive clustering method with default parameters: range of influence = 0.5, squash factor = 1.25, acceptance ratio = 0.5, and rejection ratio = 0.15. The model followed a Sugeno-type structure with 30 Gaussian input membership functions, one generated if–then rule, and one linear output membership function. The number and type of membership functions were determined automatically by the algorithm. Model training was performed using a hybrid learning approach that combined the gradient descent method with the least-squares method. Training error for each iteration was calculated using the root mean square error (RMSE). The maximum number of training iterations was set to 30.

The diagnostic performance was measured using the area under the ROC curve (AUC), sensitivity (Se), specificity (Sp), positive predictive value (PPV), and negative predictive value (NPV). Agreement between raters as well as between the developed models and physician-based nutritional risk assessment was measured using Cohen’s Kappa coefficient (κ).

The developed models were compared using predefined criteria that included diagnostic performance and practical applicability. Diagnostic performance was assessed using sensitivity (Se), specificity (Sp), area under the ROC curve (AUC), positive predictive value (PPV), negative predictive value (NPV), and Cohen’s kappa coefficient (κ) in relation to the physician-based subjective malnutrition risk assessment and the SGNA tool.

To improve interpretability and clinical relevance, models were also compared descriptively with respect to the number of predictors, the transparency of the decision structure, and computational efficiency (training time and model complexity), where applicable. Models demonstrating high AUC, balanced Se and Sp, strong agreement (κ), and lower practical complexity were considered to have a comparative advantage. Se and Sp were interpreted using predefined cut-offs (good: both ≥ 80%; fair: one < 80% but both > 50%; poor: one ≤ 50%), and κ values were interpreted according to established thresholds [4,47,48].

Although this hold-out validation approach provided an initial estimate of model performance on independent data, additional resampling-based internal validation procedures for overall model performance assessment, such as k-fold cross-validation or bootstrap resampling, were not performed and should be considered in future studies to further assess model stability and reduce optimism in performance estimates.

McNemar’s exact test was used to assess marginal homogeneity between each model and SGNA tool; p-values were obtained via the exact two-sided binomial test on the discordant pairs [49]. Agreement was quantified with Cohen’s κ with 95% asymptotic (Wald) confidence intervals based on the large-sample variance of κ; intervals were truncated to the admissible range (−1, 1) [50,51].

## 3. Results

A total of 180 children were enrolled in the study between May and October 2021, recruited from six pediatric wards: nephrology and hypertension, gastroenterology and nutrition, general pediatrics, neurology, pulmonology and allergology, and child and adolescent psychiatry. The cohort had a median age of 120.6 months (95% CI: 108–142; range 1–216), with 86 (47.8%) males and 94 (52.2%) females. In the development phase (May–July 2021), 142 children were included (median age 123 months, 95% CI: 93–138), with 69 (48.6%) males and 73 (51.4%) females. In the validation phase (September–October 2021), 38 children were assessed (median age 143 months, 95% CI: 113–169), including 17 (44.7%) males and 21 (55.3%) females (Table S2, Supplementary Material S4).

Differences in anthropometric indicators between children classified as not at nutritional risk and those at nutritional risk are presented in Table S3 (Supplementary Material S4). Mean z-scores for WFA, BMI, MUAC, and TSF were significantly lower in children at nutritional risk compared with those not at risk (all *p* < 0.001). In contrast, no significant difference was observed for HFA between the two groups (*p* = 0.304).

Overall, the results showed that advanced machine-learning and evolutionary models achieved the highest diagnostic performance, whereas simpler decision-tree models offered greater transparency and bedside feasibility. Across validation analyses, the GP, ANN, and ANFIS models demonstrated the strongest agreement with the physician-based subjective malnutrition risk assessment and the best overall discrimination. By contrast, decision-tree models showed lower diagnostic accuracy but remained clinically relevant because of their simplicity and interpretability. These findings indicate a practical trade-off between predictive performance and ease of implementation in routine pediatric ward settings.

### 3.1. Development of Nutritional Screening Models

Diagnostic performance was evaluated using AUC, sensitivity (Se), specificity (Sp), positive predictive value (PPV), negative predictive value (NPV), and Cohen’s κ, with higher values indicating better discrimination and agreement.

Responses from the questionnaire (*n* = 277 variables) were analyzed against the subjective malnutrition risk assessment using chi-squared tests. This process yielded 144 variables significantly associated with risk of malnutrition, which were subsequently reduced to the 30 most relevant predictors based on a Mean Decrease Accuracy > 2 (Table S4, Supplementary Material S5) [5]. These predictors were incorporated into the development of predictive models.

The Decision Tree (Gini) model contained only a single root node with two terminal leaves (Figure S3 in Supplementary Material S6). The Decision Tree (Information Gain) model contained 1 branching node, 3 internal nodes, and 4 terminal leaves (Figure S4 in Supplementary Material S6). The Decision Tree (CHAID) model contained three levels, four internal nodes, and five terminal leaves (Figure S5 in Supplementary Material S6).

The Random Forest (optimized) model included 30 predictor variables (Table S4 in Supplementary Material S5). Variable importance, assessed by the mean decrease in Gini coefficient, identified “Loss of MUSCLE MASS in the quadriceps: medial depression/atrophy and/or prominent knees” as the strongest predictor (Mean decrease in Gini = 10.682, selected 579 times). The importance of the remaining variables was lower (Figure S6 in Supplementary Material S7).

The XGBoost model was developed with 30 input variables using logistic regression as the objective function. Variable selection during model training resulted in 18 variables being retained (Table S6 and Figure S7 in Supplementary Material S8).

The Lasso model (Table S7 in Supplementary Material S9) included 12 of the 30 variables. The optimal λ determined by cross-validation was 0.04902633. The most important predictor was “Loss of MUSCLE MASS in the quadriceps: medial depression/atrophy and/or prominent knees”.

The GP system evolved an optimal GP model (Figure S8 in Supplementary Material S10) in generation 90 (seed 67), consisting of 471 nodes with a depth of 27 and 20 terminal genes. The model produced real-valued predictions of malnutrition risk, using a threshold of 0 for classification, and was generated with 200 independent starts (evolutions) in 23 min on an Intel i7 processor.

The Matlab ANN model (Figure S2, Supplementary Material S3) reached the stopping criterion after an average of six iterations, with a total training time of only a few seconds. In the ANN model, the internal connections between inputs and outputs are inaccessible to the user, limiting detailed interpretation and reducing the model’s practical usefulness.

The Neural network (basic) model included all 30 variables, whereas the Neural network (optimized) model was developed with only the eight most significant variables (X1–X8) (Table S8 in Supplementary Material S11). The Neural network (optimized) model is illustrated in Figure S9 in Supplementary Material S11, showing the connections between the eight input variables, the hidden layer, and the two output classes (at risk of malnutrition/not at risk of malnutrition). The numerical weights of the network are provided in Table S9 in Supplementary Material S11.

The Sugeno-type ANFIS model was successfully generated with 30 Gaussian input membership functions, one rule, and one linear output function (Figures S10 and S11, Supplementary Material S12). Training stopped after two iterations, well before the maximum of 30, with a total training time of 3–5 s. The output vector (142 × 1) of the trained model ranged from −0.2387 to +1.3040.

A synthesized comparison of the diagnostic performance of all developed models is presented in Table S10 (Supplementary Material S13), while criterion and concurrent validity results are summarized in Tables S11 and S12 (Supplementary Material S13), respectively.

As shown in Table S10 in Supplementary Material S13, the developed models generally demonstrated good diagnostic performance, with most models achieving Se and Sp above approximately 80% or higher. Agreement with the subjective malnutrition risk assessment (κ = 0.62–1.00) was classified as substantial to almost perfect. The GP and ANFIS models also demonstrated almost perfect agreement (κ = 0.811–1.00).

The GP model demonstrated the best validation results (AUC = 1.000, Se = 100%, Sp = 100%, κ = 1.00, 95% CI 1.00–1.00), correctly identifying all children and adolescents at risk of malnutrition (TP = 15) as well as all without risk (TN = 23). Similarly, the ANN (AUC = 0.978, Se = 100%, Sp = 95.7%, κ = 0.95, 95% CI 0.82, 1.00) and ANFIS (AUC = 0.923, Se = 93.3%, Sp = 91.3%, κ = 0.84, 95% CI 0.63, 1.00) models showed very high diagnostic accuracy.

The remaining models also demonstrated good performance, with AUC values ranging from 0.813 to 0.978. The lowest number of correctly identified children and adolescents without nutritional risk (TN = 19) was observed in the Decision Tree (Gini), Decision Tree (Information Gain), and Decision Tree (CHAID) models.

From a translational perspective, these findings suggest that GP, ANN, and ANFIS may represent promising candidates for future integration into digital decision-support tools, provided that their performance is confirmed in external, multicenter, and prospective studies. In contrast, decision-tree models may be more feasible for future bedside screening applications because of their transparency and ease of use, although their clinical utility also requires further validation.

Regarding the number of variables included, five models incorporated all 30 variables, while the GP model used 20 variables, XGBoost 18, Lasso 12, and the optimized neural network 8. The smallest number of variables was found in the Decision Tree (Gini) (*n* = 1), followed by the Decision Tree (Information Gain), and the Decision Tree (CHAID), each with three variables.

In the validation analyses, the subjective malnutrition risk assessment served as the primary reference standard, while SGNA was used for concurrent validity comparisons.

#### 3.1.1. Criterion Validity

The developed models were tested with 38 children and demonstrated good overall performance (Table S11 in Supplementary Material S13). Agreement with the subjective malnutrition risk assessment was substantial to almost perfect across most models. The GP model showed the highest agreement (κ = 1.00, 95% CI 1.00–1.00), indicating almost perfect concordance, while the ANN model also achieved excellent agreement (κ = 0.95, 95% CI 0.84–1.00). The ANFIS model followed closely with κ = 0.84 (95% CI 0.66–1.00).

Compared with the WHO classification of nutritional status, the κ values ranged from 0.51 to 0.78, corresponding to moderate to substantial agreement. The GP and ANN models again performed best, with κ = 0.78 (95% CI 0.58–0.98) and κ = 0.84 (95% CI 0.82, 1.00), respectively. The ANFIS model also agreed substantially (κ = 0.84, 95% CI 0.63, 1.00).

Differences in AUC values between the developed models and the WHO reference classification were generally small and statistically non-significant. The GP model outperformed the WHO classification (ΔAUC = −0.110, DeLong *p* = 0.037), suggesting higher discriminative ability. Other models, including decision trees, random forests, XGBoost, and Lasso regression, showed substantial agreement but did not significantly differ from the reference methods.

Together, these findings confirm the criterion validity of the GP and ANN models, which demonstrated the highest diagnostic accuracy and strongest agreement with both the subjective malnutrition risk assessment and WHO classification.

#### 3.1.2. Concurrent Validity

The results (Table S12, Supplementary Material S13) show that simpler models, such as decision trees (Gini, Information Gain, CHAID) demonstrated only substantial agreement with SGNA (κ = 0.67–0.79), indicating acceptable but lower consistency. Lasso regression and the basic neural network also fell within this range. In contrast, more advanced and optimized models, including Random forests, XGBoost, GP, Neural network (optimized), ANN, and ANFIS, achieved almost perfect agreement (κ ≥ 0.83), with high Se, Sp, PPV, and NPV. McNemar’s test was non-significant across all comparisons (*p* > 0.05), suggesting no systematic differences in classification between SGNA and the evaluated models.

## 4. Discussion

Our study is the first to develop a pediatric screening model for malnutrition using a wide range of machine learning and evolutionary computation methods, including decision trees, random forests, XGBoost, ANN, ANFIS, and genetic programming (GP). By doing so, it directly addresses the current gap in pediatric nutritional screening, where available tools either rely mainly on anthropometry or on simple rule-based scores with heterogeneous and sometimes suboptimal diagnostic performance [4].

The main findings demonstrate that GP, ANN, and ANFIS models achieved the highest performance in this sample, with very high agreement (κ ≥ 0.81), whereas simpler models (decision trees) showed lower accuracy but provided great interpretability, making them easier to implement in clinical practice. Validation in an independent cohort (*n* = 38) further confirmed the robustness of the models, which rely solely on non-invasive and clinically applicable indicators. Although the validation sample was relatively small, the consistent performance across multiple modelling approaches strengthens confidence in the robustness and generalizability of the observed patterns.

Currently available pediatric screening tools (e.g., STRONGkids, STAMP, PYMS, and others) are limited by heterogeneity in reference standards and cut-off values, as well as by validation, making it difficult to compare directly the tools or identify a universally “best” one among them [4,20,52]. For hospitalized children, effective nutritional screening necessitates instruments that achieve both high Se and Sp [20].

In this context, the diagnostic performance of our models compares favorably with that reported for commonly used screening tools. When evaluated against a full dietetic assessment (i.e., subjective malnutrition risk assessment), our models—specifically GP model, ANN model, ANFIS model, and XGBoost model—demonstrated high Se (80–100%), Sp (82.6–100%), PPV (75–100%), and NPV (86.4–100%), with predominantly substantial to almost perfect agreement (κ = 0.62–1.00). In contrast, published studies on PYMS and STAMP report wide variability in Se (47.6–95.7%) and Sp (63.8–94.9%), frequently accompanied by low PPV (31–58%) despite high NPV (>90%), and only fair to moderate agreement (κ = 0.28–0.57) [4,53,54,55,56,57,58]. This imbalance limits their clinical utility, particularly in accurately identifying children genuinely at nutritional risk.

A similar pattern emerges when SGNA is used as the comparator. In our study, advanced models, particularly GP, ANN, ANFIS, optimized random forest, and XGBoost, retained high diagnostic performance against SGNA, with Se ranging from 85.7% to 100%, Sp from 87.5% to 95.8%, PPV from 82.4% to 92.3%, and NPV from 91.7% to 100%, and almost perfect agreement for most advanced models (κ = 0.83–0.89). These results compare favorably with previously published screening tools validated against SGNA, such as SCAN or PNST, which often demonstrate very high Se but at the expense of low Sp (39–82%), leading to a higher rate of false positives and reduced clinical precision [4]. Overall, our findings indicate that the proposed models provide a more balanced and clinically meaningful trade-off between Se and Sp than existing pediatric screening instruments.

Overall, our findings indicate that models based on machine learning and evolutionary computation may compare favorably with previously published approaches for developing pediatric screening tools by providing a more balanced and clinically meaningful combination of diagnostic accuracy (Se, Sp, AUC, κ) and practical utility. Importantly, they are consistent with the current ASPEN, ESPEN, and ESPGHAN recommendations, all of which highlight the importance of early and accurate nutritional screening [21,22,23].

A possible explanation for the superior performance of advanced models such as GP, ANN, and ANFIS lies in their ability to capture complex, non-linear interactions among multiple clinical indicators, which are not adequately represented in traditional rule-based screening tools. Pediatric malnutrition is a multifactorial condition influenced by a combination of anthropometric, clinical, and functional factors; therefore, models capable of integrating these heterogeneous inputs are more likely to achieve higher diagnostic accuracy. In contrast, simpler models, such as decision trees, rely on a limited number of variables and hierarchical splits, which improve transparency and interpretability but may reduce sensitivity to subtle or early manifestations of malnutrition.

From a practical perspective, these differences highlight an important trade-off between predictive performance and usability. In our comparison, simpler interpretable models (decision trees, lasso) offered greater transparency and bedside feasibility, whereas more complex models (random forest, XGBoost, ANN, ANFIS, and GP) generally achieved higher diagnostic performance. While advanced models may be promising candidates for future integration into electronic decision-support systems, simpler models may be more readily implemented in routine clinical workflows without the need for technological infrastructure. This suggests that a combined approach, in which simple models are used for initial screening and more complex models for confirmatory assessment, may represent the most effective strategy for real-world clinical application.

In developing these models, we incorporated performance recommendations consistent with established usability criteria [28], models should be: rapid (so as not to burden routine workflow), simple (without complex calculations or specialized knowledge), non-invasive (avoiding painful or demanding procedures), cost-effective (not dependent on expensive equipment or laboratory tests), feasible for ward-level use (applicable in everyday clinical settings rather than only in research environments), and require minimal staff training. Decision trees are visually transparent, require no computational resources or technological support (e.g., software, tablets), and can be rapidly applied in everyday clinical practice. In contrast, more complex models such as GP, ANN, and ANFIS could be implemented as computer programs or mobile applications, like the approaches taken with the PeDiSMART tool, CWNST tool, or STRONGkids application [59,60,61]. Such computer-integrated models could be embedded into existing clinical software already used by healthcare professionals to support the treatment and care of hospitalized children. Evidence demonstrated that the implementation of electronic screening tools improves patient care, reduces the error rate associated with paper-based formats, and has the potential to enhance the identification of children at risk [62].

In this study, the subjective malnutrition risk assessment performed by pediatric specialists was selected as the primary reference standard because no gold standard diagnostic assessment exists. This decision reflects its role as the clinical gold standard, since it combines anthropometric data, medical history, and expert clinical judgment, enabling a more comprehensive evaluation than structured tools alone. In contrast, many published screening tools (STRONGkids, STAMP, SCAN, PHaM, CWNST, NEST, and PNST) have been developed or validated against anthropometric criteria based on the WHO classification system (BMI, MUAC, WFA, HFA, and WFH) [18,20,55,60,63,64,65,66,67,68]. These criteria, however, capture only a partial aspect of our reference standard and overlook the broader assessment of a child’s nutritional status. Although anthropometry is a useful, non-invasive, and widely applied method, it should be complemented with clinical and dietary information to ensure accurate diagnosis and adequate nutrition [69]. While indispensable for nutritional assessment, anthropometry alone is insufficient for individualized patient care, particularly in clinical settings. At the population level, it provides valuable indicators of nutritional problems, whereas individual evaluations require integration with a wider clinical context [69,70].

Nevertheless, the chosen reference standards also have important limitations that should be acknowledged when interpreting the results. The physician-based subjective malnutrition risk assessment, although clinically comprehensive, inevitably includes an element of professional judgment and may therefore be influenced by assessor experience and interpretation [4,21]. Although inter-rater agreement in our study was substantial to almost perfect, some degree of subjectivity cannot be fully excluded. Similarly, SGNA is a structured and validated tool, but it also incorporates subjective clinical appraisal and does not represent a universally accepted gold standard for pediatric malnutrition diagnosis [4,71]. The absence of a single universally accepted reference standard in pediatric malnutrition research has important implications: model performance may vary depending on the comparator used, and apparently high agreement may partly reflect alignment with the chosen reference framework rather than definitive diagnostic truth. These considerations support cautious interpretation of the results and further underscore the need for external validation and future studies using broader multicenter reference frameworks.

Our results highlight significant differences depending on the reference standard applied. When using the SGNA tool as the comparator, the models achieved moderately high diagnostic performance, confirming the usefulness of SGNA as a validated and widely applied tool in pediatric populations. These findings are consistent with previous validation studies; for instance, Ong et al. [72] reported moderate to high diagnostic performance of the SGNA tool compared with objective nutritional parameters, underscoring its validity as an assessment tool. The SGNA tool in our study did not fully capture the breadth of clinical characteristics associated with nutritional risk, which was reflected in lower AUC, Se, and Sp values relative to the results obtained with the subjective malnutrition risk assessment. In the latter, our models demonstrated higher diagnostic accuracy (AUC, κ) and identified a different number of children with a risk of malnutrition. Taken together, these results suggest that comprehensive assessments better reflect the complexity of clinical nutritional status and contribute more effectively to the accurate detection of at-risk children, whereas SGNA remains an important but limited screening tool. Previous studies similarly demonstrated moderately high diagnostic accuracy of SGNA, supporting its role as a useful, though not comprehensive, method for identifying pediatric malnutrition [72,73].

In the present validation sample, the GP model showed the highest apparent diagnostic accuracy, correctly identifying all children at risk of malnutrition. This suggests promising discriminatory performance in this dataset, although its robustness and clinical role require confirmation in larger external studies. However, it should be noted that GP relies on a relatively large number of variables (*n* = 20), which limits its practicality as a bedside screening instrument. Screening tools need to be rapid, simple, and feasible with a minimal number of indicators [28]; therefore, we propose the GP model primarily as an assessment tool, which can complement screening approaches and provide a more comprehensive clinical evaluation when screening indicates increased risk.

This study has several limitations. First, the sample size was relatively modest, particularly for the independent validation set, which may limit the stability of performance estimates. In addition, although the inclusion of children aged 1 month to 18 years reflects routine pediatric ward practice in our institution, this broad age range introduces substantial clinical and developmental heterogeneity. Infants, children, and adolescents differ in nutritional physiology, growth trajectories, disease profiles, and the interpretation of anthropometric and clinical indicators.

Consequently, a larger and more representative sample would be needed to support stronger generalizability of the proposed models across the full pediatric age spectrum. The required sample size was calculated based on prevalence and the same prospectively recruited cohort and was subsequently used for the development and validation of the screening models reported in the present study. Consequently, no separate a priori sample size calculation based on expected Se and Sp was performed as the original study design focused on prevalence estimation. As a result, the validation sample was small (*n* = 38), which constrains statistical power and leads to wider CIs. In addition, the questionnaire-derived predictors were not subjected to formal pilot testing with test–retest or internal-consistency reliability assessment prior to the main study, which may affect the reproducibility of some questionnaire-based items.

Pilot testing with formal reliability evaluation is planned in future prospective validation studies. Although blinding procedures and consistent effects across methods are reassuring, these limitations warrant cautious interpretation of the findings. Second, although the model was evaluated on a hold-out validation set, no external dataset was available for assessment of generalizability beyond the source population. In addition, external validation in larger, multicenter cohorts is necessary before clinical implementation. Accordingly, the model’s reported performance—while encouraging—should be interpreted with caution, as perfect discrimination metrics may indicate a residual risk of overfitting despite the applied model-complexity controls. Third, the study was conducted in a single healthcare system, which may restrict the applicability of the findings to other clinical environments. Future work will include external validation in additional Slovenian hospitals and larger, more heterogeneous cohorts to confirm the robustness and clinical utility of the proposed model [4,5]. Fourth, some models (e.g., GP) include high number of variables, which may reduce their feasibility for rapid bedside application, despite their excellent diagnostic accuracy. Fifth, the models were validated only in general pediatric wards; further testing in diverse clinical settings, such as higher-risk intensive care or pediatric oncology, is needed to evaluate their robustness and applicability across different patient populations. A sixth limitation is that the study did not include neonates younger than 1 month, who are treated in separate neonatal units and have distinct nutritional needs and growth references.

As a result, the developed models are not directly generalizable to neonatal populations, and future research should address the development or adaptation of screening tools specifically for this age group. Finally, although the reference standard was a comprehensive physician-based subjective malnutrition risk assessment, no universally accepted gold standard for pediatric malnutrition exists, which represents an inherent limitation in validation research. An additional limitation is that model calibration was not assessed in this study. Given the small validation sample (*n* = 38), formal calibration analyses (e.g., calibration plots or Brier score) were not performed due to potential instability. This is particularly relevant for more complex models that may be prone to overfitting. Future studies should include calibration assessment in larger independent cohorts.

The development of user-friendly digital applications could facilitate bedside use and enhance the clinical utility of the proposed tools. Moreover, integrating these models into electronic decision-support systems may further streamline nutritional risk screening, reduce user error, and enable timely intervention in hospitalized children.

## 5. Conclusions

This single-center study developed and validated several non-invasive pediatric malnutrition screening models using machine learning and evolutionary computation methods. Across diverse approaches, including decision trees, random forests, XGBoost, lasso regression, artificial neural networks (ANN), ANFIS, and genetic programming (GP), most models demonstrated promising to high diagnostic performance within this single-center sample when compared with physician-based subjective malnutrition risk assessment and the SGNA tool. The GP model showed the highest apparent diagnostic accuracy in this dataset, whereas ANN and ANFIS also demonstrated high performance using fewer variables, highlighting their potential for future screening applications, pending further validation.

The models proposed in this study address key limitations of existing pediatric malnutrition screening tools, which often rely on anthropometric measurements, subjective evaluations, or rigid rule-based algorithms that may inadequately detect early or subtle manifestations of malnutrition. By integrating multiple non-invasive clinical indicators and capturing complex relationships between them, the proposed models provide a flexible and precise approach to nutritional risk prediction. They are conceptually aligned with ASPEN, ESPEN, and ESPGHAN recommendations for early screening and may be suitable for future implementation by nurses, physicians, and dietitians after adequate external and prospective validation. In such contexts, they could potentially be applied at hospital admission (or within the first 24 h), with repeat screening performed weekly throughout the hospital stay, thereby supporting standardized screening across clinical settings.

While advanced models such as GP, ANN, and ANFIS showed the highest performance in this sample and appear promising for future integration into electronic decision-support systems, simpler models, such as decision trees, demonstrated lower accuracy but greater transparency and feasibility for bedside use. These findings support a complementary approach, where advanced models may serve as assessment tools and simpler models as rapid screening approaches, but both require further validation before routine clinical use.

Study limitations include the single-center design, the relatively small validation sample, the broad pediatric age range with associated clinical heterogeneity, and the lack of external validation, all of which may limit generalizability. Future studies should therefore prioritize external validation in larger, multicenter cohorts and prospective evaluation of clinical impact, including screening timeliness, referral to nutritional assessment, and clinical outcomes.

This study suggests that machine learning and evolutionary computation approaches can provide promising, non-invasive, and potentially clinically feasible screening models for pediatric malnutrition risk, representing a meaningful advancement over existing rule-based tools. By integrating multiple clinical indicators and modelling their complex interactions, the proposed models offer improved diagnostic performance while maintaining applicability in routine clinical settings. Together, these models may eventually support earlier and more consistent identification of children at risk of malnutrition, thereby facilitating timely nutritional interventions and improved clinical outcomes, provided that their performance is confirmed in broader external validation studies. However, external validation in larger, multicenter cohorts is necessary before these models can be implemented in routine clinical practice.

Importantly, advanced models such as GP, ANN, and ANFIS achieved the highest diagnostic performance and appear promising for future integration into electronic decision-support systems, whereas simpler models, such as decision trees, may be more appropriate for rapid bedside screening due to their transparency and ease of use. This supports a scalable and flexible implementation strategy that can be adapted to different clinical environments and levels of available technological infrastructure.

## Data Availability

The data supporting this study’s findings are available from the corresponding author upon reasonable request.

## Supplementary Materials

The following supporting information can be downloaded at: https://www.mdpi.com/article/10.3390/children13050617/s1, Figure S1: Visual diagram of phase 1 and 2 of developing of nutritional screening models; Table S1: Inter-rater reliability in the evaluation of nutritional risk using a subjective malnutrition risk assessment performed by a pediatric specialist or resident (6 or 2 nutritional categories); Figure S2: Topology of ANN (artificial neural network) model for predicting risk of malnutrition; Table S2: Characterization of the study sample; Table S3: Differences in nutritional evaluated data among nutritional risk categories; Table S4: Variables with Mean Decrease Accuracy metric value greater than 2; Figure S3: Decision tree (Gini) model; Figure S4: Decision tree (Information Gain) model; Figure S5: Decision tree (CHAID) model; Table S5: Included variables in the Random forest and Random forest (optimized) model with the number of variable selections in internal nodes and the values of the mean decrease in Gini; Figure S6: Variables included into the optimized Random Forest model, ranked according to their mean decrease in Gini coefficient values; Table S6: List of variables in the XGBoost model; Figure S7: Variables included in the XGBoost model according to the Gain attribute; Table S7: Included variables in the Lasso model; Figure S8: Equation of the GP model; Table S8: Calculated variable importance based on the χ^2^ test; Figure S9: Topology of ANN (artificial neural network) model for predicting risk of malnutrition; Table S9: Optimized Neural Network Model Weights with Eight Input Variables; Figure S10: Structure of the ANFIS model for predicting risk of malnutrition; Figure S11: FIS system with Gaussian membership function applied; Table S10: Measures of Diagnostic Accuracy of Models Developed with Data Mining Methods and Non-Invasive Indicators; Table S11: Evaluation of developed models based on agreement with the subjective malnutrition risk assessment, WHO classification of nutritional status, and the selected statistical models (Cohen’s kappa statistic, DeLong test, McNemar test); Table S12: Comparison of the developed screening models with the SGNA tool.

## Abbreviations

The following abbreviations are used in this manuscript:

WHO	World Health Organization
ASPEN	American Society for Parenteral and Enteral Nutrition
AND	Academy of Nutrition and Dietetics
CWNST	Children’s Wisconsin Nutrition Screening Tool
ESPEN	European Society for Clinical Nutrition and Metabolism
ESPGHAN	European Society for Paediatric Gastroenterology, Hepatology and Nutrition
SGNA	Subjective Global Nutritional Assessment
NEST	The Nutrition Evaluation Screening Tool
SPNRS	Simple Pediatric Nutritional Risk Score
STAMP	Screening Tool for the Assessment of Malnutrition in Paediatrics
PYMS	Paediatric Yorkhill Malnutrition Score
PeDiSMART	Pediatric Digital Scaled MAlnutrition Risk screening Tool
PNST	Paediatric Nutrition Screening Tool
STRONG_kids_	Screening Tool for Risk on Nutritional status and Growth
SCAN	Nutrition Screening tool for childhood Cancer
PHaM	Risk Score to Predict Nutritional Deterioration in Hospitalized Pediatric Patients
W	Weight
H	Height
L	Length
MUAC	Mid-Upper Arm Circumference
BMI	Body mass index
WFH/L	Weight for Height/Length
HFA	Height for Age
WFA	Weight for Age
CDC	Centers for Disease Control and Prevention
ROC	Receiver Operating Characteristic
AUC	Area Under the Curve
Se	Sensitivity
Sp	Specificity
PPV	Positive Predictive Value
NPV	Negative Predictive Value
TN	True Negative
TP	True Positive
κ	Cohen’s kappa agreement index
XGBoost	eXtreme Gradient Boosting
CHAID	Chi-square Automatic Interaction Detection
ANN	Artificial Neural Networks
ANFIS	Adaptive Neuro-Fuzzy Inference System
GP	Genetic Programming
CI	Confidence Interval
%	Percent
n	Number
v	Variables
Lasso	Least absolute shrinkage and selection operator
DL	DeLong test
SD	Standard deviation
MSE	Mean squared error
RMSE	Root mean square error
ID3	Information Gain/ID3 algorithm
χ^2^	Chi-square
