# Supplementary material for: Development and Validation of Non-Invasive Machine-Learning Screening Models for Pediatric Malnutrition in Hospitalized Children: A Single-Center Study
Source: Children (Basel). 2026 Apr 29;13(5):617. doi: 10.3390/children13050617 (PMC13204657; doi:10.3390/children13050617)
Supplement: Supplementary file 1 [file children-13-00617-s001.zip › children-4254447-supplementary.pdf]

Supplementary Material S1.

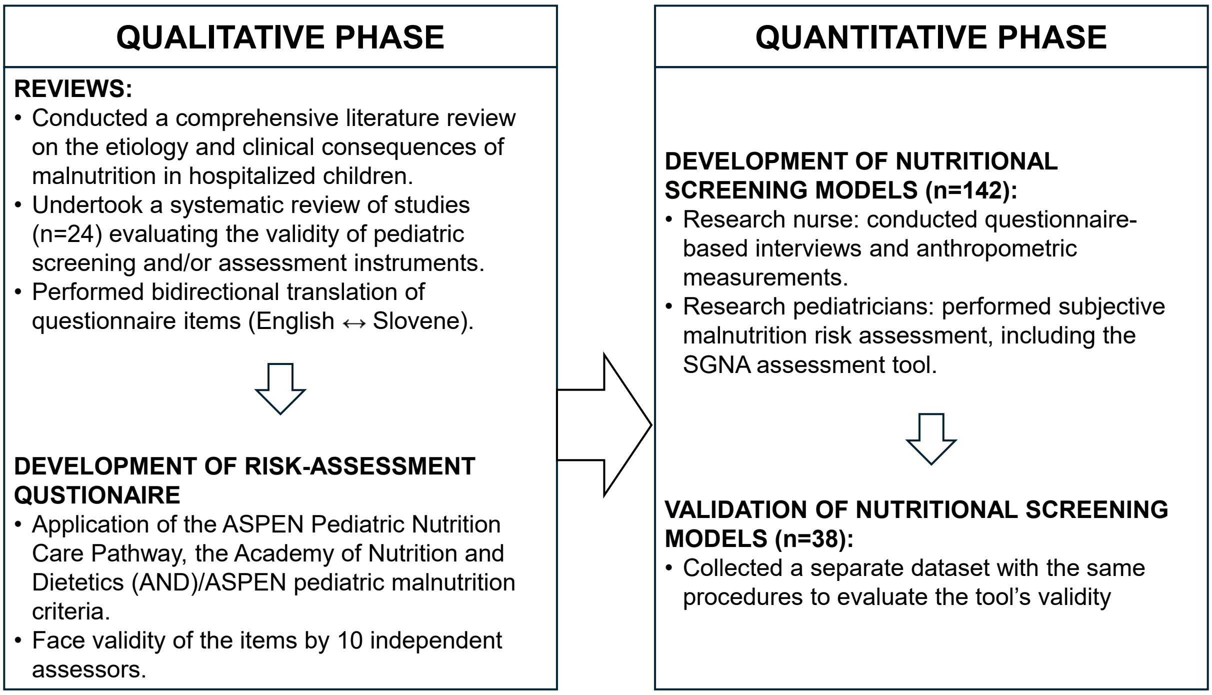

Figure S1. Visual diagram of phase 1 and 2 of developing of nutritional screening models

Supplementary Material S2.

Table S1. Inter-rater reliability in the evaluation of nutritional risk using a subjective malnutrition risk assessment performed by a pediatric specialist or resident (6 or 2 nutritional categories).

Subjective malnutrition risk assessment performed by pediatricians		n	κ [95 % CI]	κ[a]
Evaluator 1	Evaluator 2	28	1.0 [1.0, 1.0]	pe
Evaluator 1	Evaluator 3	37	0.9 [0.7, 1.0]	pe
Evaluator 2	Evaluator 3	15	0.7 [0.4, 1.0]	su

Legend: CI = confidence interval, n = number of hospitalized children included, κ = kappa coefficient of agreement, κ[a] = evaluation criteria for kappa: almost perfect agreement (pe) = κ from 0.81 to 1.00, substantial agreement (su) = κ from 0.61 to 0.80, % = percentage.

Supplementary Material S3.

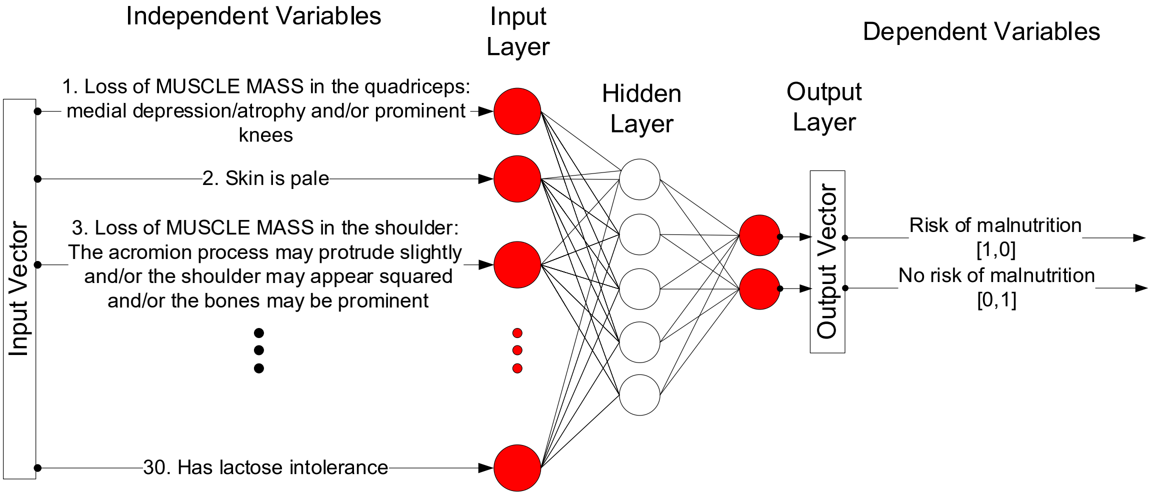

Figure S2. Topology of ANN (artificial neural network) model for predicting risk of malnutrition.

Supplementary Material S4.

Table S2. Characterization of the study sample.

	Involved in the study (n=180)	Development phase (n=142)	Validation phase (n=38)
**Male:Female n (%)**	86 (47.8):94 (52.2)	69 (48.6):73 (51.4)	17 (44.7):21 (55.3)
**Age in months (Median (95% CI))**	130 (108, 142)	123 (93, 138)	143 (113, 169)
**Prevalence of malnutrition:**	**n (%)**	**n (%)**	**n (%)**
**Total malnutrition** [a]	69 (38.4)	54 (38)	15 (39.5)
**Absence of malnutrition** [a]	111 (61.6)	88 (62)	23 (60.5)
**BMI** [b, c]	**n (%)**	**n (%)**	**n (%)**
**Total malnutrition**	46 (34.9)	35 (28.8)	11 (28.9)
**Absence of malnutrition**	134 (74.5)	107 (75.4)	27 (71.1)

Legend: [a] = by physician-based subjective malnutrition risk assessment; [b] = defined by Becker et al. and as recommended by the ASPEN and Academy of Nutrition and Dietetics [34]; [c] = defined by WHO.

Table S3. Differences in nutritional evaluated data among nutritional risk categories

	Overall (n=180) mean ± SD	Not at risk (n=111) mean ± SD	At risk (n=69) mean ± SD	Mean difference ± SE (95 % CI)	t^d	*p*-value
**z score WFA**	0.19 ± 1.40	0.81 ± 1.18	−0.82 ± 1.11	1.63 ± 0.17 (1.29–1.97)	9.36	<0.001
**z score HFA**	0.39 ± 1.29	0.47 ± 1.25	0.26 ± 1.36	0.21 ± 0.20 (-0.19, 0.61)	1.03	0.304
**z score BMI**	-0.10 ± 1.54	0.71 ± 1.08	-1.39 ± 1.25	2.10 ± 0.18 (1.74, 2.46)	11.51	<0.001
**z score MUAC**	-0.36 ± 1.37	0.33 ± 1.09	-1.48 ± 0.98	1.80 ± 0.16 (1.49, 2.12)	11.49	<0.001
**z score TSF**	-1.32 ± 1.50	-0.58 ± 1.31	-2.41 ± 1.03	1.83 ± 0.18 (1.47, 2.19)	10.06	<0.001

Legend: WFA = Weight for Age; HFA = Height/length for Age; BMI = Body mass index; MUAC = Mid-Upper Arm Circumference; TSF = Triceps skin-fold thickness; 95 % CI = 95% confidence interval; n = number; [d] = Welch t-test value.

Supplementary Material S5.

Table S4. Variables with Mean Decrease Accuracy metric value greater than 2.

Variables (n = 30)	Mean Decrease Accuracy (RF)
Loss of MUSCLE MASS in the quadriceps: medial depression/atrophy and/or prominent knees.	12.74294654
Skin is pale.	12.54599404
Loss of MUSCLE MASS in the shoulder: the acromion process may protrude slightly and/or the shoulder may appear squared and/or the bones may be prominent.	10.85744344
Neck is thin: noticeably reduced fat under the chin, neck structures clearly visible.	9.399246742
Loss of SUBCUTANEOUS FAT under the eyes: hollowed look, depressions, dark circles.	9.247257535
Loss of SUBCUTANEOUS FAT on the ribs is evident, but indentations are not pronounced.	8.972809942
Loss of MUSCLE MASS in the scapula/ribs: bones are prominent with significant depressions [between the ribs or around the shoulder blade].	7.353547494
Loss of MUSCLE MASS in the clavicle: bone is protruding/prominent.	6.431115652
He/she had poor weight gain over the last few months [past 3 months].	5.918259554
Nutrient intake is adequate, without changes; eating as usual, not losing weight, growing and developing normally.	5.767338789
Loss of SUBCUTANEOUS FAT on the triceps: some depth of fat tissue present, but not ample; skin appears loose-fitting.	4.421929285
Even a minor blow can cause a bruise.	4.314094268
No recent weight loss, as body weight is static.	4.147506546
Food refusal is present.	4.072897314
Nutrient intake is suboptimal for solid foods.	3.962858296
There is rejection of sweet (sugary) foods, confectionery.	3.576241627
Laxatives are needed to empty the bowels.	3.559247276
Symptoms are present that affect oral food intake (e.g., pain in eating, anorexia, vomiting, nausea, dysphagia, diarrhoea, dental problems, feels full quickly or constipation).	3.417919746
Early satiety is present, leading to reduced oral food intake.	3.147423868
Food intake is less than a whole meal.	3.037764332
Skin is dry.	2.668102155
Feeding is compromised due to musculoskeletal changes or problems [e.g., injuries to hands or upper extremities; neck extension; lack of movement; scoliosis; difficulty raising hands to mouth; poor/absent gross/fine motor skills; muscle spasms; etc.].	2.46006653
NOT passing stool regularly.	2.416573708
Appetite is poor, does not consume most of the food from 3 meals a day (or equivalent).	2.35236238
Extremes of dietary restriction are present: deep and thoughtful thinking about food and eating.	2.244228284
Has a rather low or reduced sense of hunger.	2.103629086
In the past, there were factors that affected nutrition - food intake.	2.076734596
Signs of fatigue and/or frequent sleepiness are present.	2.013365871
Has celiac disease.	2.007548252
Has lactose intolerance.	2.000946058

Legend: n = number; RF = Random Forest).

Supplementary Material S6.

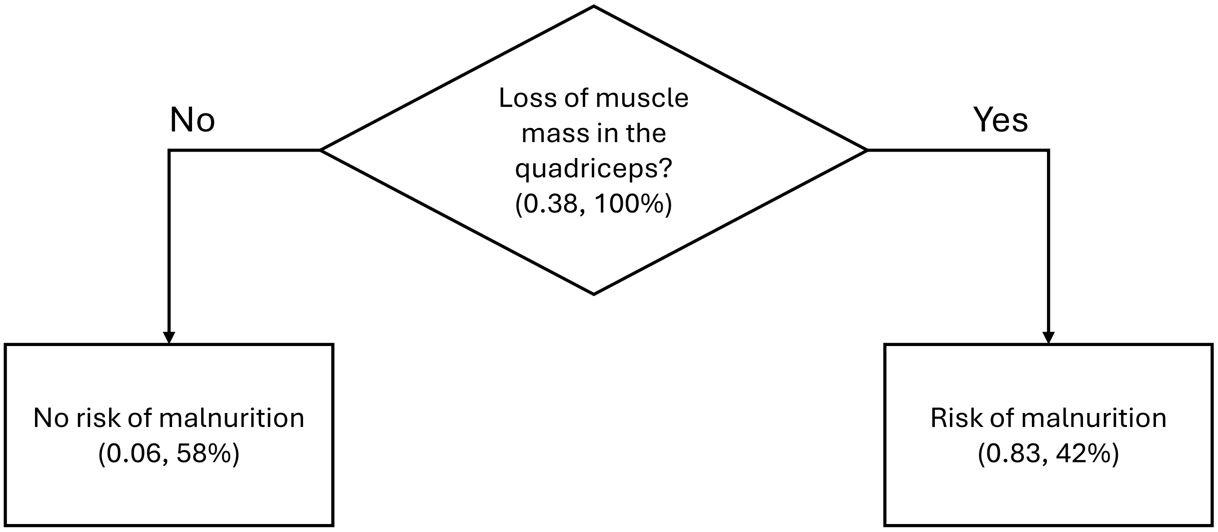

Figure S3. Decision tree (Gini) model.

Legend: In the decision tree, internal node represents the decision criterion used for classification, while the terminal nodes (leaves) indicate the final outcome (risk of malnutrition or no risk of malnutrition). Values shown in the nodes are presented as (probability, percentage), where the probability indicates the predicted likelihood of malnutrition and the percentage indicates the proportion of children assigned to the corresponding node. In the terminal nodes, the percentage reflects the proportion of children classified according to the node outcome (children at risk of malnutrition in risk leaves and children not at risk of malnutrition in no risk leaves).

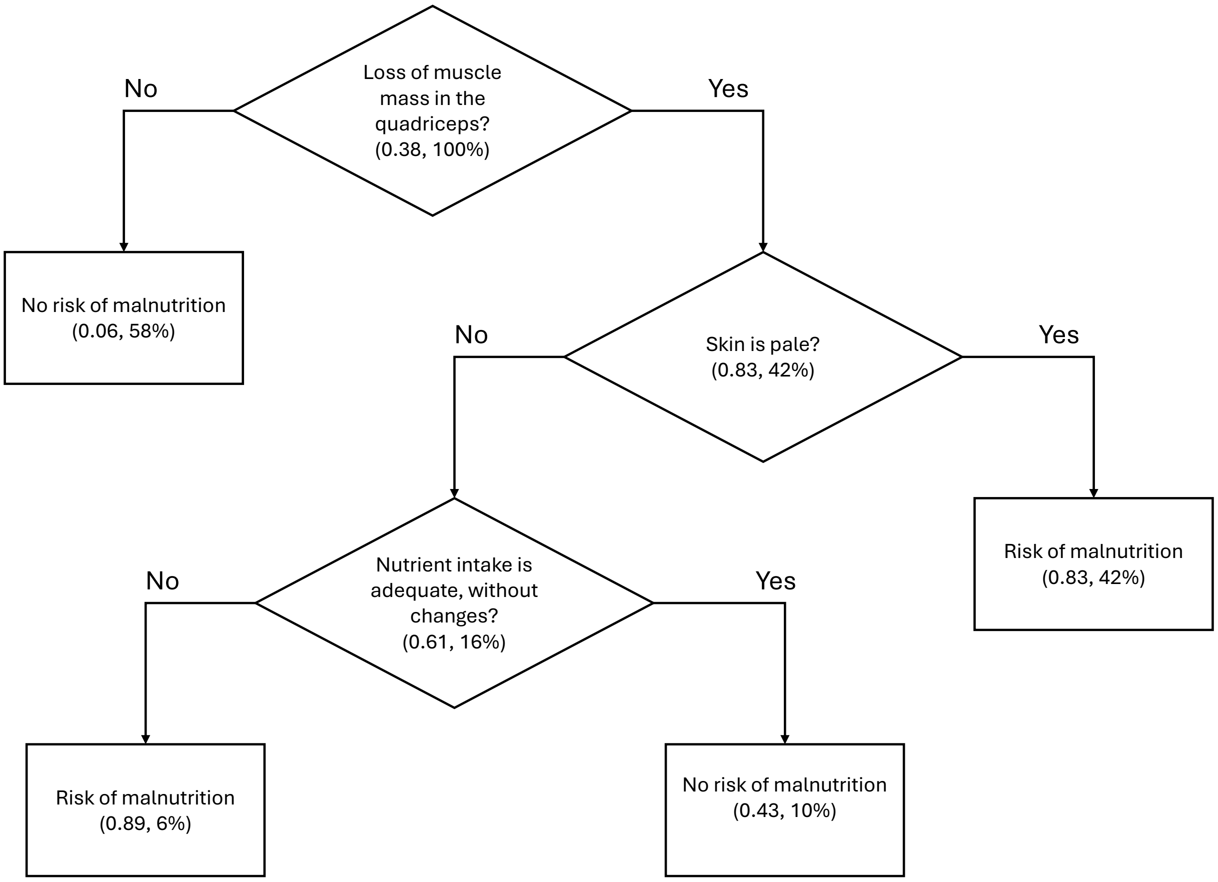

Figure S4. Decision tree (Information gain) model.

Legend: In the decision tree, internal nodes represent decision criteria, whereas terminal nodes (leaves) represent the final classification (risk of malnutrition or no risk of malnutrition). Values shown in the nodes are presented as (probability, percentage). The probability indicates the predicted likelihood of malnutrition, while the percentage indicates the proportion of children assigned to that node. In internal nodes, the percentage represents the proportion of children at risk of malnutrition within that node. In terminal nodes, the percentage reflects the proportion of children classified according to the node outcome, i.e., children at risk of malnutrition in risk leaves and children not at risk of malnutrition in no risk leaves.

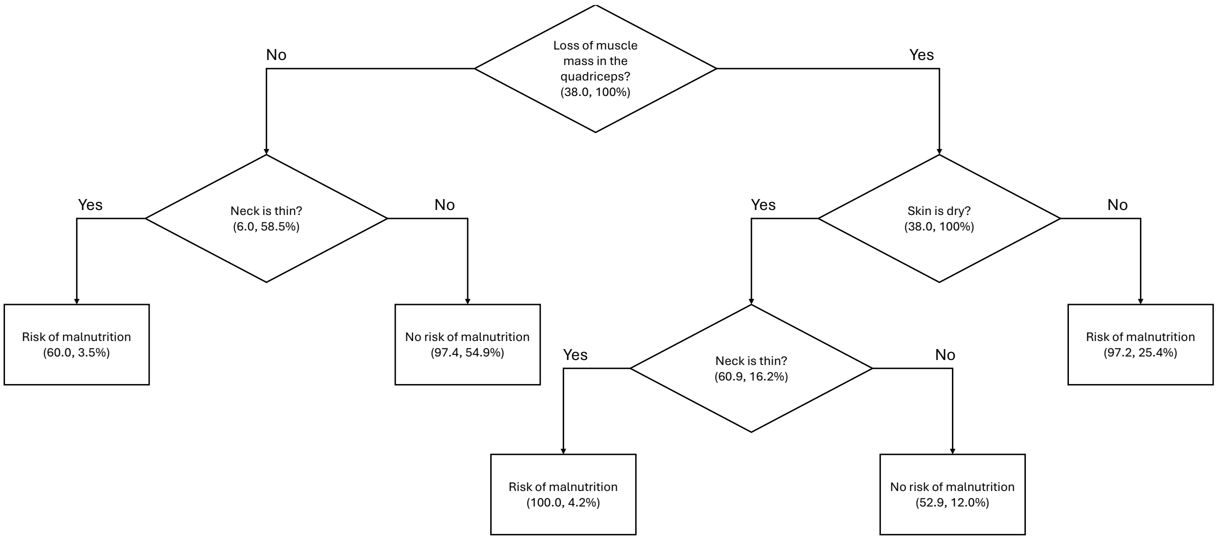

Figure S5. Decision tree (CHAID) model.

Legend: In the decision tree, internal nodes represent decision criteria, whereas terminal nodes (leaves) represent the final classification (risk of malnutrition or no risk of malnutrition). Values shown in the nodes are presented as (probability, percentage). The probability indicates the predicted likelihood of malnutrition, while the percentage indicates the proportion of children assigned to that node. In internal nodes, the percentage represents the proportion of children at risk of malnutrition within that node. In terminal nodes, the percentage reflects the proportion of children classified according to the node outcome, i.e., children at risk of malnutrition in risk leaves and children not at risk of malnutrition in no risk leaves.

Supplementary Material S7.

Table S5. Included variables in the Random forest and Random forest (optimized) model with the number of variable selections in internal nodes and the values of the mean decrease in Gini

Variables (n = 30)	Number of variable selections	Mean decrease in Gini
Loss of MUSCLE MASS in the quadriceps: medial depression/atrophy and/or prominent knees.	579	10.682
Skin is pale.	589	6.905
Loss of MUSCLE MASS in the shoulder: the acromion process may protrude slightly and/or the shoulder may appear squared and/or the bones may be prominent.	488	6.444
Loss of SUBCUTANEOUS FAT on the ribs is evident, but indentations are not pronounced.	420	4.767
Loss of MUSCLE MASS in the scapula/ribs: bones are prominent with significant depressions [between the ribs or around the shoulder blade].	470	3.873
Loss of SUBCUTANEOUS FAT under the eyes: hollowed look, depressions, dark circles.	376	3.835
Neck is thin: noticeably reduced fat under the chin, neck structures clearly visible.	387	3.289
Loss of SUBCUTANEOUS FAT on the triceps: some depth of fat tissue present, but not ample; skin appears loose-fitting.	416	2.63
Loss of MUSCLE MASS in the clavicle: bone is protruding/prominent.	363	2.313
He/she had poor weight gain over the last few months [past 3 months].	399	2.103
Food refusal is present.	395	1.672
Nutrient intake is adequate, without changes; eating as usual, not losing weight, growing and developing normally.	354	1.589
Symptoms are present that affect oral food intake (e.g., pain in eating, anorexia, vomiting, nausea, dysphagia, diarrhoea, dental problems, feels full quickly or constipation).	353	1.576
Nutrient intake is suboptimal for solid foods.	331	1.427
Food intake is less than a whole meal.	311	1.289
There is rejection of sweet (sugary) foods, confectionery.	226	1.083
No recent weight loss, as body weight is static.	364	1.078
Laxatives are needed to empty the bowels.	227	0.927
Early satiety is present, leading to reduced oral food intake.	282	0.899
In the past, there were factors that affected nutrition - food intake.	320	0.762
NOT passing stool regularly.	286	0.743
Appetite is poor, does not consume most of the food from 3 meals a day (or equivalent).	285	0.711
Signs of fatigue and/or frequent sleepiness are present.	283	0.66
Even a minor blow can cause a bruise.	143	0.558
Has lactose intolerance.	250	0.519
Skin is dry.	92	0.401
Has a rather low or reduced sense of hunger.	139	0.366
Has celiac disease.	111	0.273
Feeding is compromised due to musculoskeletal changes or problems [e.g., injuries to hands or upper extremities; neck extension; lack of movement; scoliosis; difficulty raising hands to mouth; poor/absent gross/fine motor skills; muscle spasms; etc.]	69	0.243

Variables (n = 30)	Number of variable selections	Mean decrease in Gini
Extremes of dietary restriction are present: deep and thoughtful thinking about food and eating.	36	0.106

Legend: n = number.

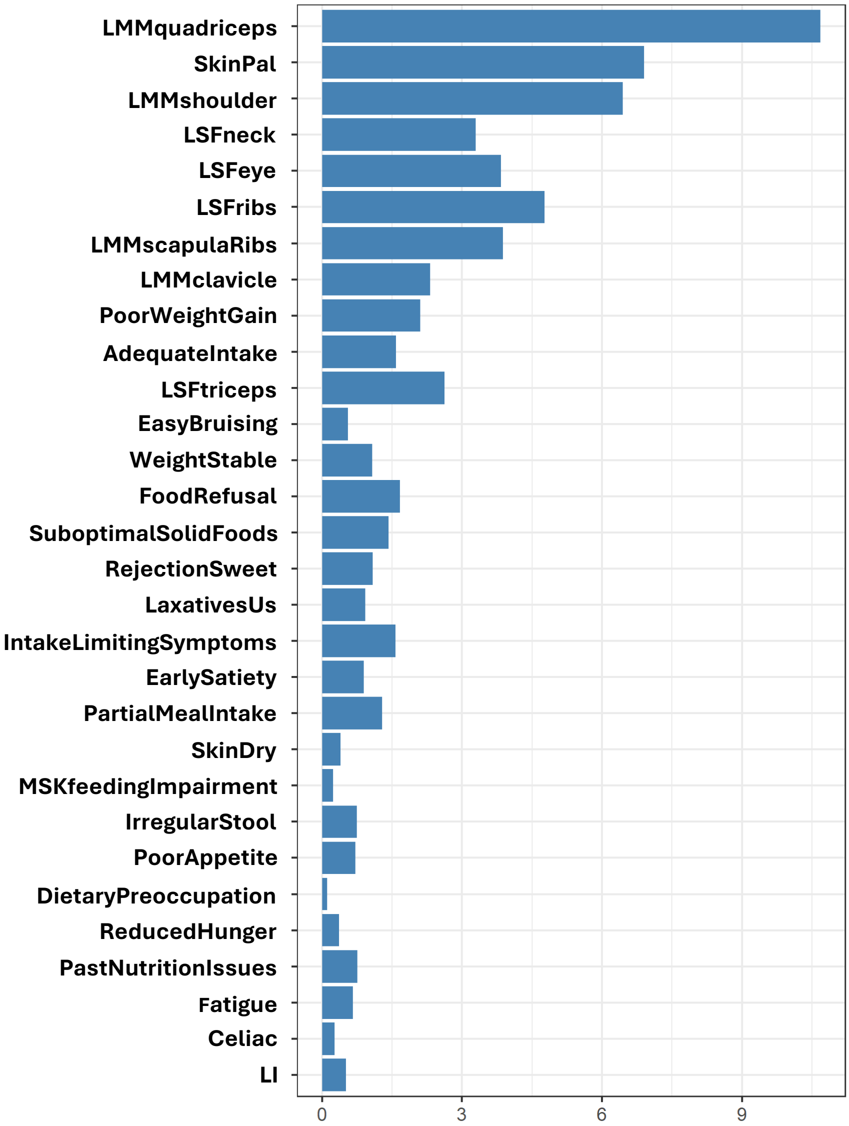

Figure S6. Variables included into the optimized Random Forest model, ranked according to their mean decrease in Gini coefficient values.

Legend: LMMquadriceps = Loss of MUSCLE MASS in the quadriceps: medial depression/atrophy and/or prominent knees; SkinPale = Skin is pale; LMMshoulder = Loss of MUSCLE MASS in the shoulder: the acromion process may protrude slightly and/or the shoulder may appear squared and/or the bones may be prominent; LSFneck = Neck is thin: noticeably reduced fat under the chin, neck structures clearly visible; LSFeye = Loss of SUBCUTANEOUS FAT under the

eyes: hollowed look, depressions, dark circles; LSFribs = Loss of SUBCUTANEOUS FAT on the ribs is evident, but indentations are not pronounced; LMMscapulaRibs = Loss of MUSCLE MASS in the scapula/ribs: bones are prominent with significant depressions [between the ribs or around the shoulder blade]; LMMclavicle = Loss of MUSCLE MASS in the clavicle: bone is protruding/prominent; PoorWeightGain = He/she had poor weight gain over the last few months [past 3 months]; AdequateIntake = Nutrient intake is adequate, without changes; eating as usual, not losing weight, growing and developing normally; LSFtriceps = Loss of SUBCUTANEOUS FAT on the triceps: some depth of fat tissue present, but not ample; skin appears loose-fitting; EasyBruising = Even a minor blow can cause a bruise; WeightStable = No recent weight loss, as body weight is static; FoodRefusal = Food refusal is present; SuboptimalSolidFoods = Nutrient intake is suboptimal for solid foods; RejectionSweet = There is rejection of sweet (sugary) foods, confectionery; LaxativeUs = Laxatives are needed to empty the bowels; IntakeLimitingSymptoms = Symptoms are present that affect oral food intake (e.g., pain in eating, anorexia, vomiting, nausea, dysphagia, diarrhoea, dental problems, feels full quickly or constipation); EarlySatiety = Early satiety is present, leading to reduced oral food intake; PartialMealIntake = Food intake is less than a whole meal; SkinDry = Skin is dry; MSKfeedingImpairment; IrregularStool = NOT passing stool regularly; PoorAppetite = Appetite is poor, does not consume most of the food from 3 meals a day (or equivalent); DietaryPreoccupation = Extremes of dietary restriction are present: deep and thoughtful thinking about food and eating; ReducedHunger = Has a rather low or reduced sense of hunger; PastNutritionIssues = In the past, there were factors that affected nutrition - food intake; Fatique = Signs of fatigue and/or frequent sleepiness are present; Celiac = Has celiac disease; LI = Has lactose intolerance.

Supplementary Material S8.

Table S6. List of variables in the XGBoost model

Variables (n = 18)	Gini	Cover
Loss of MUSCLE MASS in the quadriceps: medial depression/atrophy and/or prominent knees.	0.266207	0.145784
Skin is pale.	0.156996	0.152418
Loss of MUSCLE MASS in the shoulder: the acromion process may protrude slightly and/or the shoulder may appear squared and/or the bones may be prominent.	0.138919	0.088433
Loss of SUBCUTANEOUS FAT on the ribs is evident, but indentations are not pronounced.	0.13841	0.087477
Loss of MUSCLE MASS in the scapula/ribs: bones are prominent with significant depressions [between the ribs or around the shoulder blade].	0.117133	0.121614
Food refusal is present.	0.06226	0.096048
Loss of SUBCUTANEOUS FAT under the eyes: hollowed look, depressions, dark circles.	0.037328	0.046723
Loss of SUBCUTANEOUS FAT on the triceps: some depth of fat tissue present, but not ample; skin appears loose-fitting.	0.035492	0.045327
Nutrient intake is adequate, without changes; eating as usual, not losing weight, growing and developing normally.	0.009152	0.056799
He/she had poor weight gain over the last few months [past 3 months].	0.008966	0.026972
Symptoms are present that affect oral food intake (e.g., pain in eating, anorexia, vomiting, nausea, dysphagia, diarrhoea, dental problems, feels full quickly or constipation).	0.00837	0.025058
Appetite is poor, does not consume most of the food from 3 meals a day (or equivalent).	0.008251	0.017003
Nutrient intake is suboptimal for solid foods.	0.003213	0.037743
Neck is thin: noticeably reduced fat under the chin, neck structures clearly visible.	0.002948	0.004709
NOT passing stool regularly.	0.00205	0.013668
Food intake is less than a whole meal.	0.001959	0.00893
Early satiety is present, leading to reduced oral food intake.	0.001738	0.016673
Has lactose intolerance.	0.00061	0.00862

Legend: n = number.

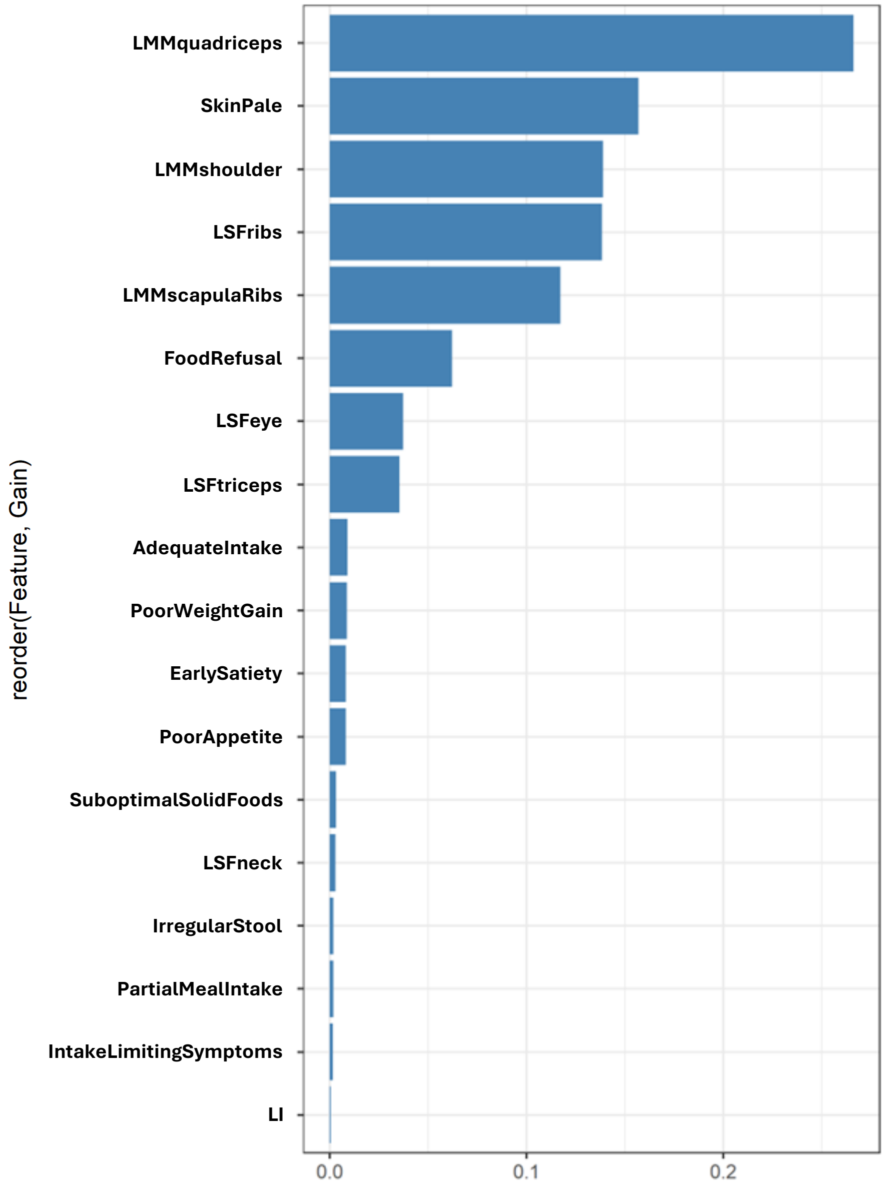

Figure S7. Variables included in the XGBoost model according to the Gain attribute.

Legend: LMMquadriceps = Loss of MUSCLE MASS in the quadriceps: medial depression/atrophy and/or prominent knees; SkinPale = Skin is pale; LMMshoulder = Loss of MUSCLE MASS in the shoulder: the acromion process may protrude slightly and/or the shoulder may appear squared and/or the bones may be prominent; LSFribs = Loss of SUBCUTANEOUS FAT on the ribs is evident, but indentations are not pronounced; LMMscapulaRibs = Loss of MUSCLE MASS in the scapula/ribs: bones are prominent with significant depressions [between the ribs or around the shoulder blade]; FoodRefusal = Food refusal is present; LSFeye = Loss of SUBCUTANEOUS FAT under the eyes: hollowed look, depressions, dark circles; LSFtriceps = Loss of SUBCUTANEOUS FAT on the triceps: some depth of fat tissue present, but not ample; skin appears loose-fitting; AdequateIntake = Nutrient intake is adequate, without changes; eating as usual, not losing weight, growing and developing normally; PoorWeightGain = He/she had poor weight gain over the last few months [past 3 months]; EarlySatiety = Early satiety is present, leading to reduced oral food intake; PoorAppetite = Appetite is poor, does

not consume most of the food from 3 meals a day (or equivalent); SuboptimalSolidFoods = Nutrient intake is suboptimal for solid foods; LSFneck = Neck is thin: noticeably reduced fat under the chin, neck structures clearly visible; IrregularStool = NOT passing stool regularly; PartialMealIntake = Food intake is less than a whole meal; IntakeLimitingSymptoms = Symptoms are present that affect oral food intake (e.g., pain in eating, anorexia, vomiting, nausea, dysphagia, diarrhoea, dental problems, feels full quickly or constipation); LI = Has lactose intolerance.

Supplementary Material S9.

Table S7. Included variables in the Lasso model

Variables (n = 12)	β
Loss of MUSCLE MASS in the quadriceps: medial depression/atrophy and/or prominent knees.	0.389777674
Skin is pale.	0.195936758
Loss of MUSCLE MASS in the shoulder: the acromion process may protrude slightly and/or the shoulder may appear squared and/or the bones may be prominent.	0.077203468
Neck is thin: noticeably reduced fat under the chin, neck structures clearly visible.	0.136453876
Loss of SUBCUTANEOUS FAT under the eyes: hollowed look, depressions, dark circles.	0.038612606
Loss of SUBCUTANEOUS FAT on the ribs is evident, but indentations are not pronounced.	0
Loss of MUSCLE MASS in the scapula/ribs: bones are prominent with significant depressions [between the ribs or around the shoulder blade].	0.023974011
Loss of MUSCLE MASS in the clavicle: bone is protruding/prominent.	0
He/she had poor weight gain over the last few months [past 3 months].	0.027025407
Nutrient intake is adequate, without changes; eating as usual, not losing weight, growing and developing normally.	0
Loss of SUBCUTANEOUS FAT on the triceps: some depth of fat tissue present, but not ample; skin appears loose-fitting.	0.002380398
Even a minor blow can cause a bruise.	0.003083445
No recent weight loss, as body weight is static.	0
Food refusal is present.	0.024265164
There is rejection of sweet (sugary) foods, confectionery.	0
Nutrient intake is suboptimal for solid foods.	0.071878989
Laxatives are needed to empty the bowels.	0.007679528
Symptoms are present that affect oral food intake (e.g., pain in eating, anorexia, vomiting, nausea, dysphagia, diarrhoea, dental problems, feels full quickly or constipation).	0
Early satiety is present, leading to reduced oral food intake.	0
Food intake is less than a whole meal.	0
Skin is dry.	0
Feeding is compromised due to musculoskeletal changes or problems [e.g., injuries to hands or upper extremities; neck extension; lack of movement; scoliosis; difficulty raising hands to mouth; poor/absent gross/fine motor skills; muscle spasms; etc.]	0
NOT passing stool regularly.	0
Appetite is poor, does not consume most of the food from 3 meals a day (or equivalent).	0
Extremes of dietary restriction are present: deep and thoughtful thinking about food and eating.	0
Has a rather low or reduced sense of hunger.	0
In the past, there were factors that affected nutrition - food intake.	0
Signs of fatigue and/or frequent sleepiness are present.	0
Has celiac disease.	0
Has lactose intolerance.	0

Legend: n = number; β = beta coefficient.

Supplementary Material S10.

$$\text{Risk of malnutrition} = \text{Fatique} - \text{IrregularStool} + \text{LI} + \text{LMMquadriceps} + \text{LMMshoulder} + \text{LSFeye} +$$

$$\text{LSFribs} + \text{PartialMealIntake}\,\text{PastNutritionIssues} - \frac{2\,\text{PartialMealIntake}}{\text{SkinPale}} + \text{SkinPale} - \frac{\text{SkinPale}}{\text{LMMscapulaRibs}} +$$

$$2\,\text{SuboptimalSolidFoods} - \text{WeightStable} - \Big(\text{AdequateIntake}\Big(2\,\text{Fatique} - \text{IrregularStool} + \text{LI} +$$

$$\text{LMMquadriceps} + \text{LMMshoulder} + \text{LSFeye} + \text{LSFribs} + \text{PartialMealIntake}\,\text{PastNutritionIssues} -$$

$$\frac{2\,\text{PartialMealIntake}}{\text{SkinPale}} - \frac{\text{SkinPale}}{\text{LMMquadriceps} - \text{LMMscapulaRibs} + \text{LSFribs}} + 2\,\text{SuboptimalSolidFoods} -$$

$$(-\text{Fatique} + \text{FoodRefusal})\Big(\text{LI} + \text{LSFeye} - \frac{\text{PartialMealIntake}}{\text{SkinPale}} + \text{SkinPale} + \text{SuboptimalSolidFoods} -$$

$$\Big(-1 + \text{LI} + \text{LSFeye} + \text{LSFribs} + \text{SuboptimalSolidFoods} - \frac{4.8499\,\text{Fatique}\,\text{SuboptimalSolidFoods}}{\text{LSFribs}^2\,\text{SkinPale}} -$$

$$\text{WeightStable}\Big) \Big/ \Big(\text{MusculoskeletalChanges} - \frac{\text{PartialMealIntake}}{\text{SkinPale}} + \text{SuboptimalSolidFoods}\Big) +$$

$$\frac{1}{\text{WeightStable}}(-\text{Fatique} - \text{FoodRefusal} + \text{LMMquadriceps} + \text{LSFribs})\,\text{PartialMealIntake}\Big) - \text{WeightStable}\Big)$$

$$\Big(\Big(\text{SuboptimalSolidFoods}\Big(-\text{LI} - \text{LSFeye} + \text{LSFribs} + \Big(-\text{FoodRefusal} + \text{LMMquadriceps} - \text{LSFeye} + \text{LSFribs} +$$

$$\frac{\text{AdequateIntake}}{\text{SkinPale}} + \frac{\text{SkinPale}}{\text{LMMquadriceps} + \text{LSFribs} - \text{SkinPale}} - \frac{4.8499\,\text{SuboptimalSolidFoods}}{\text{Fatique}\,\text{LSFribs}\,\text{SkinPale}}\Big) \Big/$$

$$\Big(-\text{SkinPale} + \frac{\text{SkinPale}}{\text{Fatique}} + \text{SuboptimalSolidFoods}\Big)\Big)\Big) \Big/ (\text{Fatique}\,\text{PartialMealIntake}) -$$

$$\Big(\text{Fatique} - \text{IrregularStool} + \text{LaxativeUs} + \text{LI} + \text{LMMquadriceps} + \text{LMMshoulder} + \text{LSFeye} +$$

$$\text{LSFribs} + \text{PartialMealIntake}\,\text{PastNutritionIssues} - \frac{\text{PartialMealIntake}}{\text{SkinPale}} -$$

$$\frac{\text{SkinPale}}{\text{LMMquadriceps} - \text{LMMscapulaRibs} + \text{LSFribs}} + \text{SuboptimalSolidFoods} - \text{WeightStable} -$$

$$(-\text{Fatique} + \text{FoodRefusal})\Big(\text{LI} + \text{LSFeye} - \frac{\text{PartialMealIntake}}{\text{SkinPale}} + \text{SuboptimalSolidFoods} - \Big(-1 + \text{LI} + \text{LSFeye} +$$

$$\text{LSFribs} + \text{SuboptimalSolidFoods} - \frac{4.8499\,\text{SuboptimalSolidFoods}}{\text{Fatique}\,\text{LSFribs}\,\text{SkinPale}} - \text{WeightStable}\Big) \Big/$$

$$\Big(\text{LSFeye} - \frac{\text{PartialMealIntake}}{\text{SkinPale}} - \frac{\text{SkinPale}}{\text{MusculoskeletalChanges}} + \text{SuboptimalSolidFoods}\Big) + (-\text{Fatique} -$$

$$\text{FoodRefusal} + \text{LMMquadriceps} + \text{LSFribs})\Big(-\text{IrregularStool} + \text{LI} + \text{LMMquadriceps} + \text{LSFeye} +$$

$$\text{LSFribs} - \text{WeightStable} - \frac{\text{SkinPale}}{-\frac{\text{PartialMealIntake}}{\text{SkinPale}} + \text{SuboptimalSolidFoods} + \text{WeightStable}}\Big)\Big)\Big) \Big/$$

$$(\text{MusculoskeletalChanges}\,\text{SkinDry})\Big)\Big) \Big/ \Big(\text{LMMquadriceps} + \text{LSFribs} - \frac{\text{PartialMealIntake}}{\text{IrregularStool}} - \text{PoorWeightGain}\Big)$$

Figure S8. Equation of the GP model

Legend: LMMquadriceps = Loss of MUSCLE MASS in the quadriceps: medial depression/atrophy and/or prominent knees; SkinPale = Skin is pale; LMMshoulder = Loss of MUSCLE MASS in the shoulder: the acromion process may protrude slightly and/or the shoulder may appear squared and/or the bones may be prominent; LSFribs = Loss of SUBCUTANEOUS FAT on the ribs is evident, but indentations are not pronounced; LMMscapulaRibs: bones are prominent with significant depressions [between the ribs or around the shoulder blade]; LSFeye = Loss of SUBCUTANEOUS FAT under the eyes: hollowed look, depressions, dark circles; PoorWeightGain = He/she had poor weight gain over the last few months [past 3 months]; FoodRefusal = Food refusal is present; AdequateIntake = Nutrient intake is adequate, without changes; eating as usual, not losing weight, growing and developing normally; SuboptimalSolidFoods = Nutrient intake is suboptimal for solid foods; PartialMealIntake = Food intake is less than a whole meal; RejectionSweet = There is rejection of sweet (sugary) foods, confectionery; WeightStable = No recent weight loss, as body weight is static; LaxativeUs = Laxatives are needed to empty the bowels; PastNutritionIssues = In the past, there were factors that affected nutrition - food intake; IrregularStool = NOT passing stool regularly; Fatique = Signs of fatigue and/or frequent sleepiness are present; LI = Has lactose intolerance; SkinDry = Skin is dry; MSKFeedingImpairment = Feeding is compromised due to musculoskeletal changes or problems [e.g., injuries to hands or upper extremities; neck extension; lack of movement; scoliosis; difficulty raising hands to mouth; poor/absent gross/fine motor skills; muscle spasms; etc.].

Supplementary Material S11.

Table S8. Calculated variable importance based on the χ^2 test

ID	Variables (n = 30)	$\chi 2$	p
X1	Loss of MUSCLE MASS in the quadriceps: medial depression/atrophy and/or prominent knees.	83.583	< 0.001
X3	Loss of MUSCLE MASS in the shoulder: the acromion process may protrude slightly and/or the shoulder may appear squared and/or the bones may be prominent.	69.184	< 0.001
X2	Skin is pale.	63.302	< 0.001
X6	Loss of SUBCUTANEOUS FAT on the ribs is evident, but indentations are not pronounced.	59.233	< 0.001
X5	Loss of SUBCUTANEOUS FAT under the eyes: hollowed look, depressions, dark circles.	54.603	< 0.001
X7	Loss of MUSCLE MASS in the scapula/ribs: bones are prominent with significant depressions [between the ribs or around the shoulder blade].	49.468	< 0.001
X4	Neck is thin: noticeably reduced fat under the chin, neck structures clearly visible.	48.902	< 0.001
X8	Loss of MUSCLE MASS in the clavicle: bone is protruding/prominent.	40.909	< 0.001
X11	Loss of SUBCUTANEOUS FAT on the triceps: some depth of fat tissue present, but not ample; skin appears loose-fitting.	40.360	< 0.001
X9	He/she had poor weight gain over the last few months [past 3 months].	37.306	< 0.001
X18	Symptoms are present that affect oral food intake (e.g., pain in eating, anorexia, vomiting, nausea, dysphagia, diarrhoea, dental problems, feels full quickly or constipation).	28.922	< 0.001
X10	Nutrient intake is adequate, without changes; eating as usual, not losing weight, growing and developing normally.	27.487	< 0.001
X15	Nutrient intake is suboptimal for solid foods.	26.255	< 0.001
X20	Food intake is less than a whole meal.	25.243	< 0.001
X14	Food refusal is present.	20.261	< 0.001
X19	Early satiety is present, leading to reduced oral food intake.	15.030	< 0.001
X13	No recent weight loss, as body weight is static.	13.664	< 0.001
X21	Skin is dry.	12,977	< 0.001
X12	Even a minor blow can cause a bruise.	11.169	0.001
X24	Appetite is poor, does not consume most of the food from 3 meals a day (or equivalent).	10.146	0.001
X28	Signs of fatigue and/or frequent sleepiness are present.	8.863	0.003
X25	Extremes of dietary restriction are present: deep and thoughtful thinking about food and eating.	7.648	0.006
X23	NOT passing stool regularly.	7.399	0.007
X16	There is rejection of sweet (sugary) foods, confectionery.	6.720	0.010
X26	Has a rather low or reduced sense of hunger.	5.986	0.014
X27	In the past, there were factors that affected nutrition - food intake.	5.350	0.021
X17	Laxatives are needed to empty the bowels.	5.136	0.023
X22	Feeding is compromised due to musculoskeletal changes or problems [e.g., injuries to hands or upper extremities; neck extension; lack of movement; scoliosis; difficulty raising hands to mouth; poor/absent gross/fine motor skills; muscle spasms; etc.]	1.177	0.278
X30	Has lactose intolerance.	0.304	0.581
X29	Has celiac disease.	0.000	1.000

Legenda: ID = Variable identification number; χ2 = Chi-square test, p = statistical significance p < 0,05

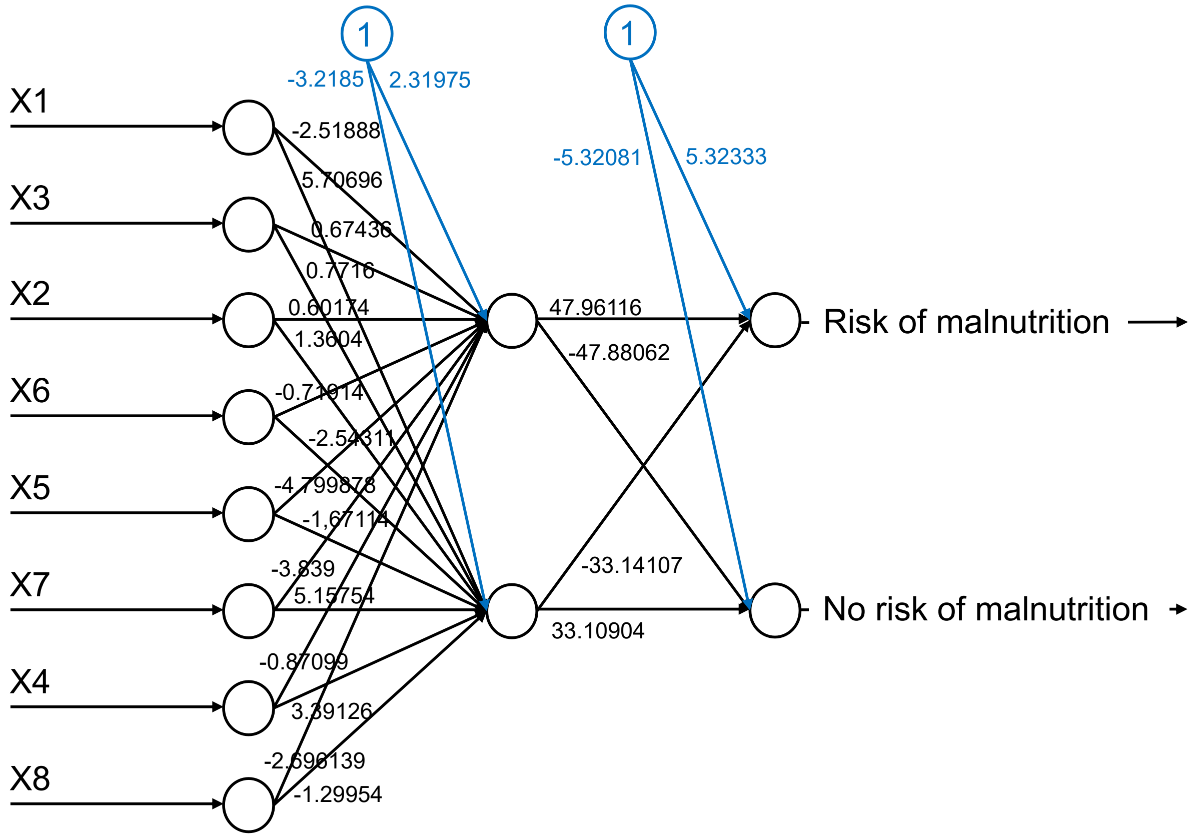

Figure S9. Topology of ANN (artificial neural network) model for predicting risk of malnutrition.

Table S9. Optimized Neural Network Model Weights with Eight Input Variables

**Weights**	**Model construction**	
**Initial weights**	**1**	**2**
1	-0.56047565	-0.4456620
2	-0.23017749	1.2240818
3	1.55870831	0.3598138
4	0.07050839	0.4007715
5	0.12928774	0.1106827
6	1.71506499	-0.5558411
7	0.46091621	1.7869131
8	-1.26506123	0.4978505
9	-0.68685285	-1.9666172
	**1**	**2**
1	0.7013559	-0.2179749
2	-0.4727914	-1.0260044
3	-1.0678237	-0.7288912
**Final weights**	**Weights between input neurons**	**Weights between neurons in the hidden layer**
1	2.3197463	-3.2185023
X1	-2.5188776	5.7069572
X3	0.6743593	0.7716629
X2	-7.6017431	1.3604111
X6	-0.7191448	-2.6431072
X5	-4.7987820	-1.6711355
X7	3.8389959	5.1575412
X4	-0.8709928	3.3912556
X8	-2.9613909	-1.2995389
	**Weights between input neurons**	**Weights between neurons in the hidden layer**
1	5.323329	-5.320813
2	47.961163	-47.880623
3	-33.141070	33.109043

Supplementary Material S12.

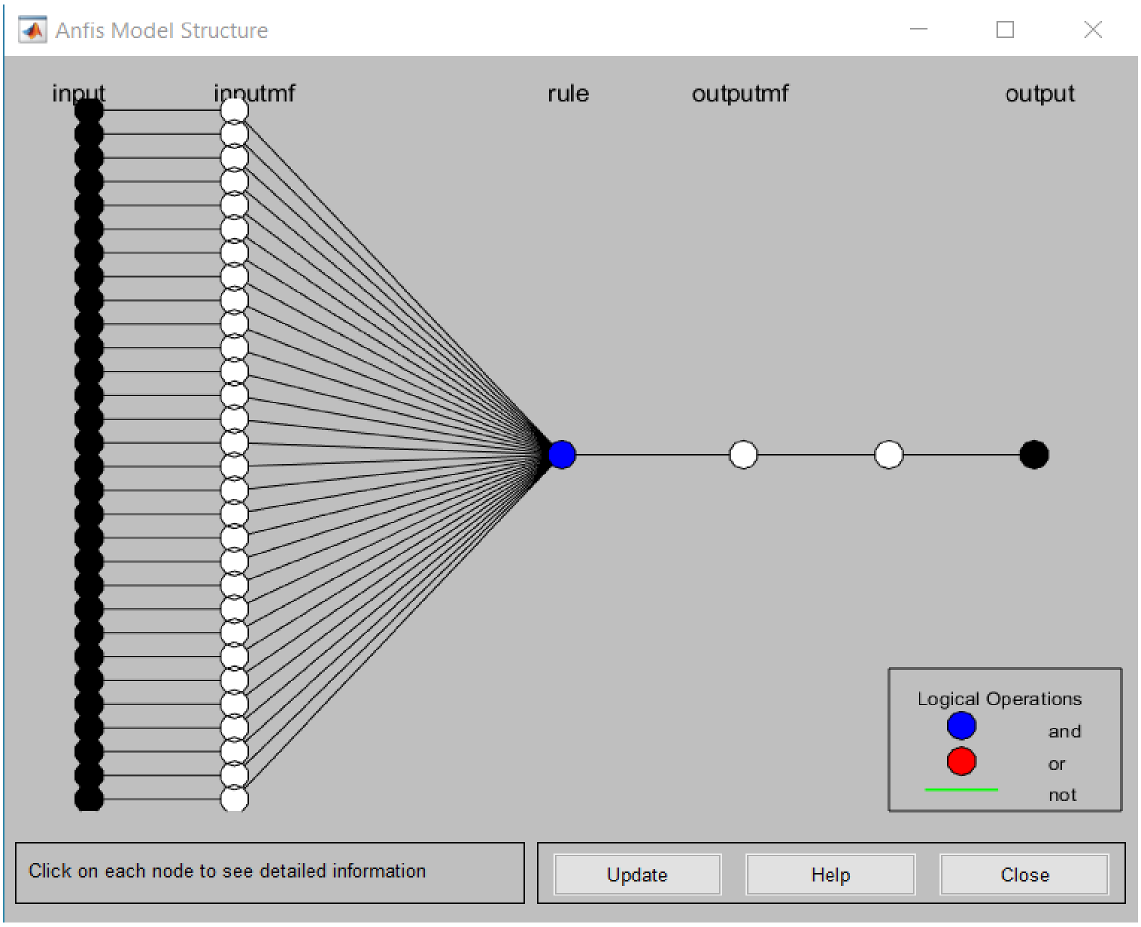

Figure S10. Structure of the ANFIS model for predicting risk of malnutrition.

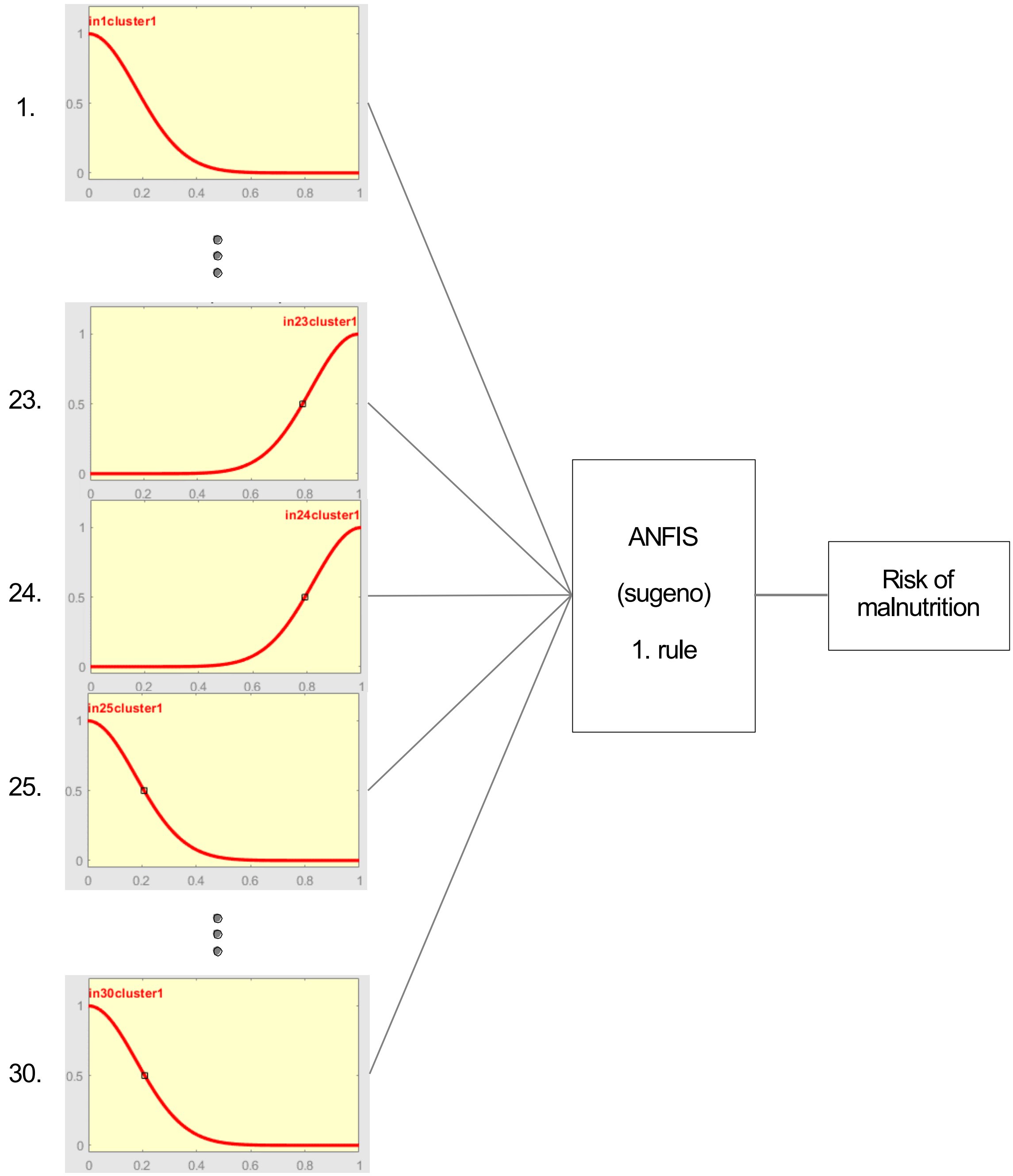

Figure S11. FIS system with Gaussian membership function applied.

Supplementary Material S13.

Table S10. Measures of Diagnostic Accuracy of Models Developed with Data Mining Methods and Non-Invasive Indicators

**Method of model development**	**Subjective malnutrition risk assessment**											
	**AUC (95% CI)**	**TP (n = 15)**	**TN (n = 23)**	**Se (%) (95 % Cl)**	**Sp (%) (95 % Cl)**	**Sp and Se[a]**	**NPV (%) (95 % Cl)**	**PPV (%) (95 % Cl)**	**Threshold**	**κ (95 % Cl)**	**κ [b]**	**Number v (n)**
WHO/CDC	0.890 (0.783, 0.997)	13	21	86.7 (59.5, 98.3)	91.3 (72.0, 98.9)	g	91.3 (72.0, 98.9)	86.7 (59.5, 98.3)	0.50	0.78 (0.54, 0.95)	su	4
Decision tree (Gini)	0.813 (0.682, 0. 944)	12	19	80 (51.9, 95.7)	82.6 (61.2, 95.0)	g	86.4 (65.1, 97.1)	75.0 (47.6, 92.7)	0.45	0.62 (0.33, 0.84)	su	1
Decision tree (Information gain)	0.850 (0.703, 0.958)	12	19	80.0 (51.9, 95.7)	82.6 (61.2, 95.0)	g	86.4 (65.1, 97.1)	75.0 (47.6, 92.7)	0.24	0.62 (0.33, 0.84)	su	3
Decision tree (CHAID)	0.880 (0.77, 0.982)	14	19	93.3 (68.1, 99.8)	82.6 (61.2, 95.0)	g	95.0 (75.1, 99.9)	77.8 (52.4, 93.6)	0.50	0.73 (0.5, 0.94)	su	3
Random forest (basic)	0.878 (0.765, 0.991)	12	22	80 (51.9, 95.7)	95.7 (78.1, 99.9)	g	88.0 (68.8, 97.5)	92.3 (64.0, 99.8)	0.50	0.77 (0.54, 0.95)	su	30
Random forest (optimized)	0.890 (0.78, 0.997)	13	21	86.7 (59.5, 98.3)	91.3 (72.0, 98.9)	g	91.3 (72.0, 98.9)	86.7 (59.5, 98.3)	0.50	0.78 (0.54, 0.95)	su	30
XGBoost	0.901 (0.805, 0.997)	14	20	93.3 (68.1, 99.8)	87.0 (66.4, 97.2)	g	95.2 (76.2, 99.9)	82.4 (56.6, 96.2)	0.45	0.78 (0.56, 0.95)	su	18
Lasso	0.868 (0.755, 0.982)	13	20	86.7 (59.5, 98.3)	87 (66.4, 97.2)	g	90.9 (70.8, 98.9)	81.2 (54.4, 96.0)	0.52	0.73 (0.48, 0.94)	su	12
GP	1.000 (1.000, 1.000)	15	23	100 (78.2, 100)	100 (85.2, 100)	g	100 (85.2, 100)	100 (78.2, 100)	0.00	1.00 (1.00, 1.00)	pe	20
Neural network (basic)	0.857 (0.736, 0.977)	12	21	80 (51.9, 95.7)	91.3 (72.0, 98.9)	g	87.5 (67.6, 97.3)	85.7 (57.2, 98.2)	0.49	0.72 (0.47, 0.93)	su	30
Neural network (optimized)	0.901 (0.805, 0.997)	14	20	93.3 (68.1, 99.8)	87.0 (66.4, 97.2)	g	95.2 (76.2, 99.9)	82.4 (56.6, 96.2)	0.37	0.78 (0.56, 0.95)	su	8
ANN	0.978 (0.936, 1.000)	15	22	100 (78.2, 100)	95.7 (78.1, 99.9)	g	100 (84.6, 100)	93.8 (69.8, 99.8)	0.32	0.95 (0.82, 1.00)	pe	30
ANFIS	0.923 (0.835,1.000)	14	21	93.3 (68.1, 99.8)	91.3 (72.0, 98.9)	g	95.5 (77.2, 99.9)	87.5 (61.7, 98.4)	0.50	0.84 (0.63, 1.00)	pe	30

Legend: n = number, % = percentage, v = variables, AUC = Area under the curve, TP = True positive, TN = True negative, Sp = Specificity, Se = Sensitivity, PPV = Positive predictive value, NPV = Negative predictive value, κ = Cohen's kappa agreement index, CI = confidence interval, [a] = Evaluation criteria for Se and Sp: good (g) = Se and Sp ≥ 80%; moderate/partial (f) = Se or Sp < 80% but both > 50%; poor (p) = Se or Sp ≤ 50%, [b] = Evaluation criteria for κ: almost perfect agreement (pe) = κ from 0.81 to 1.00, substantial agreement (su) = κ from 0.61 to 0.80, moderate agreement (m) = κ from 0.41 to 0.60, fair agreement (f) = κ from 0.21 to 0.40, slight agreement (s) = κ from 0 to 0.20, no agreement (n) = κ < 0, v = variables, WHO = World Health Organization, CDC = Centres for Disease Control and Prevention, CHAID = Chi-square Automatic Interaction Detector, GP = Genetic Programming; ANN = Artificial Neural Network, ANFIS = Adaptive Neuro-Fuzzy Inference System, Lasso = Least absolute shrinkage and selection operator, XGBoost = eXtreme Gradient Boosting.

Table S11. Evaluation of developed models based on agreement with the subjective malnutrition risk assessment, WHO classification of nutritional status, and the selected statistical models (Cohen's kappa statistic, DeLong test, McNemar test).

Method of model development	Subjective malnutrition risk assessment		WHO classification of nutritional status			
	McNemar test (n = 38)	κ (95% CI) κ[a] (n = 38)	McNemar test (n = 38)	κ (95% CI) κ[a] (n = 38)	Δ AUC (n = 38) (95 % Cl)	DL (p) (n = 38)
WHO/CDC	0.00 (p = 1.000)	0.78 (0.58, 0.98) su	/	/	/	/
Decision tree (Gini)	0.00 (p = 1.000)	0.62 (0.36, 0.87) su	0.00 (p = 1.000)	0.51 (0.23, 0.79) m	0.077 (-0.092, 0.246)	-0.894 (0.371)
Decision tree (Information gain)	0.00 (p = 1.000)	0.62 (0.36, 0. 87) su	0.00 (p = 1.000)	0.51 (0.23, 0.79) m	0.042 (-0.124, 0.208)	-0.497 (0.619)
Decision tree (CHAID)	0.80 (p = 0.371)	0.73 (0.52, 0.95) su	0.57 (p = 0.450)	0.63 (0.38, 0.88) su	0.010 (-0.131, 0.151)	-0.139 (0,890)
Random forest (basic)	0.25 (p = 0.617)	0.77 (0.57, 0.98) su	0.25 (p = 0.617)	0.77 (0.57, 0.98) su	0.012 (-0.142, 0.166)	-0.153 (0.879)
Random forest (optimized)	0.00 (p = 1.000)	0.78 (0.58, 0.98) su	0.00 (p = 1.000)	0.78 (0.58, 0.98) su	0.000 n/a (ΔAUC = 0)	0.000 (1.000)
XGBoost	0,25 (p = 0.617)	0. 78 (0.59, 0.98) su	0.17 (p = 0.683)	0.68 (0. 44, 0.91) su	-0.088 (-0.196, 0.020)	1.597 (0.110)
Lasso	0.00 (p = 1.000)	0.73 (0.51, 0. 95) su	0.00 (p = 1.000)	0.73 (0.51, 0.95) su	-0.072 (-0.188, 0.044)	1.215 (0.224)
GP	/	1.00 (1.00, 1.00) pe	0.00 (p = 1.000)	0.78 (0.58, 0,98) su	-0.110 (-0.214, -0.007)	2.084 (0.037)
Neural network (basic)	0.00 (p = 1.000)	0.72 (0.49, 0.95) su	0.00 (p = 1.000)	0.72 (0.49, 0.95) su	-0.093 (-0.201, 0.015)	1.683 (0.092)
Neural network (optimized)	0.25 (p = 0.617)	0.78 (0.59, 0.98) su	0. 17 (p = 0.683)	0.68 (0.44, 0.91) su	-0.067 (-0.188, 0.054)	1.084 (0.278)
ANN	0.00 (p = 1.000)	0.95 (0.84, 1.00) pe	0.00 (p = 1.000)	0.84 (0.66, 1.00) pe	-0.088 (-0.199, 0.023)	1.557 (0.120)
ANFIS	0.00 (p = 1.000)	0.84 (0.66, 1.00) pe	0.00 (p = 1.000)	0.73 (0.51, 0.95) su	-0.033 (-0.164, 0.098)	0.495 (0.620)

Legend: n = number, % = percentage, κ = Cohen's kappa agreement index, ΔAUC = the difference in AUC between the WHO classification of nutritional status and the evaluated model; CI = confidence interval, [a] = comparison of models with the Subjective malnutrition risk assessment, [b] = comparison of models with the WHO classification of nutritional status, DL = DeLong test, κ = Cohen's kappa agreement index, κ[a] = Evaluation

criteria for κ: almost perfect agreement (pe) = κ from 0.81 to 1.00, substantial agreement (su) = κ from 0.61 to 0.80, moderate agreement (m) = κ from 0.41 to 0.60, fair agreement (f) = κ from 0.21 to 0.40, slight agreement (s) = κ from 0 to 0.20, no agreement (n) = κ < 0.00, WHO = World Health Organization, CHAID = Chi-square Automatic Interaction Detector, GP = Genetic Programming, ANN = Artificial Neural Network, ANFIS = Adaptive Neuro-Fuzzy Inference System, Lasso = Least absolute shrinkage and selection operator, XGBoost = eXtreme Gradient Boostin, CDC = Centres for Disease Control and Prevention; n/a = non-estimable confidence intervals (CI) due to identical AUC values.

Table S12. Comparison of the developed screening models with the SGNA tool

Method of model development	SGNA										
	AUC (95 % Cl)	TP (n = 15)	TN (n = 23)	Se (%) (95 % Cl)	Sp (%) (95 % Cl)	Sp and Se[a]	NPV (%) (95 % Cl)	PPV (%) (95 % Cl)	κ (95 % Cl)	κ [b]	McNemar test (p) (n = 38)
WHO/CDC	0.866 (0.749, 0.983)	12	21	85.7 (57.2, 98.2)	87.5 (67.6, 97.3)	g	91.3 (72.0, 98.9)	86.6 (51.9, 95.7)	0.72 (0.46, 0.89)	su	0.00 (p=1.000)
Decision tree (Gini)	0.845 (0.723, 0.967)	12	20	85.7 (57.2, 98.2)	83.3 (62.6, 95.3)	g	90.9 (70.8, 98.9)	75.0 (47.6, 92.7)	0.67 (0.4, 0.89)	su	0.17 (p=0.687)
Decision tree (Information gain)	0.845 (0.723, 0.967)	12	20	85.7 (57.2, 98.2)	83.3 (62.6, 95.3)	g	86.4 (70.8, 98.9)	75.0 (47.6, 92.7)	0.67 (0.4, 0.89)	su	0.17 (p=0.687)
Decision tree (CHAID)	0.917 (0.841, 0.993)	14	20	100.0 (76.8, 100)	83.3 (62.6, 95.3)	g	100.0 (83.2, 100)	77.8 (52.4, 93.6)	0.79 (0.59, 0.95)	su	2.25 (p=0.125)
Random forest (basic)	0.908 (0.804, 1.000)	12	23	85.7 (57.2, 98.2)	95.8 (78.9, 99.9)	g	92.0 (74.0, 99.0)	92.3 (64.0, 99.8)	0.83 (0.62, 1.00)	pe	0.00 (p=1.000)
Random forest (optimized)	0.923 (0.833, 1.000)	13	22	92.9 (66.1, 99.8)	91.7 (73.0, 99.0)	g	95.7 (78.1, 99.9)	86.7 (59.5, 98.3)	0.83 (0.64, 1.00)	pe	0.00 (p=1.000)
XGBoost	0.938 (0.87, 1.000)	14	21	100 (76.8, 100)	87.5 (67.6, 97.3)	g	100.0 (83.9, 100)	82.4 (56.6, 96.2)	0.84 (0.65, 1.00)	pe	1.33 (p=0.250)
Lasso	0.902 (0.804, 0.999)	13	21	92.9 (66.1, 99.8)	87.5 (67.6, 97.3)	g	95.5 (77.2, 99.9)	81.2 (54.4, 96.0)	0.78 (0.56, 0.95.00)	su	0.25 (p=0.625)
GP	0.923 (0.833, 1.000)	13	22	92.9 (66.1, 99.8)	91.7 (73.0, 99.0)	g	95.7 (78.1, 99.9)	86.7 (59.5, 98.3)	0.83 (0.63, 1.00)	pe	0.00 (p=1.000)
Neural network (basic)	0.887 (0.776, 0.998)	12	22	85.7 (57.2, 98.2)	91.7 (73.0, 99.0)	g	91.7 (73.0, 99.0)	87.5 (57.2, 98.2)	0.77 (0.54, 0.95)	su	0.25 (p=1.000)
Neural network (optimized)	0.938 (0.87, 1.000)	14	21	100.0 (76.8, 100)	87.5 (67.6, 97.3)	g	100.0 (83.9, 100)	82.4 (56.6, 96.2)	0.84 (0.65, 1.00)	pe	1.33 (p=0.250)
ANN	0.958 (0.902, 1.000)	14	22	100.0 (76.8, 100)	91.7 (73.0, 99.0)	g	100.0 (84.6, 100)	87.5 (61.7, 98.4)	0.89 (0.72, 1.00)	pe	0.50 (p=0.500)
ANFIS	0.958 (0.902, 1.000)	14	22	100.0 (76.8, 100)	91.7 (73.0, 99.0)	g	100.0 (84.6, 100)	87.5 (61.7, 98.4)	0.89 (0.74, 1.00)	pe	0.50 (p=0.500)

Legend: n = number, % = percentage, AUC = Area under the curve, TP = True positive, TN = True negative, Sp = Specificity, Se = Sensitivity, PPV = Positive predictive value, NPV = Negative predictive value, κ = Cohen's kappa agreement index, CI = confidence interval, [a] = Evaluation criteria for Se and Sp: good (g) = Se and Sp ≥ 80%; moderate/partial (f) = Se or Sp < 80% but both > 50%; poor (p) = Se or Sp ≤ 50%, [b] = Evaluation criteria

for κ: almost perfect agreement (pe) = κ from 0.81 to 1.00, substantial agreement (su) = κ from 0.61 to 0.80, moderate agreement (m) = κ from 0.41 to 0.60, fair agreement (f) = κ from 0.21 to 0.40, slight agreement (s) = κ from 0 to 0.20, no agreement (n) = κ < 0, v = variables, WHO = World Health Organization, CDC = Centres for Disease Control and Prevention, CHAID = Chi-square Automatic Interaction Detector, GP = Genetic Programming; ANN = Artificial Neural Network, ANFIS = Adaptive Neuro-Fuzzy Inference System, Lasso = Least absolute shrinkage and selection operator, XGBoost = eXtreme Gradient Boostin.
